# A Modern Ghost Story: Increased Selective Mortality of Salmon Under Climate Extremes

**DOI:** 10.1111/gcb.70854

**Published:** 2026-04-28

**Authors:** Anna M. Sturrock, Kirsten Sellheim, Joseph Merz, Jamie Sweeney, Miranda Bell‐Tilcock, George Whitman, Kohma Arai, Malte Willmes, Carson Jeffres, Rachel C. Johnson

**Affiliations:** ^1^ School of Life Sciences University of Essex Wivenhoe Park Colchester UK; ^2^ Center for Watershed Sciences University of California Davis Davis California USA; ^3^ Cramer Fish Sciences West Sacramento California USA; ^4^ Department of Wildlife, Fish & Conservation Biology University of California Davis Davis California USA; ^5^ Delta Stewardship Council U.S. Bank Plaza Sacramento California USA; ^6^ Norwegian Institute for Nature Research Trondheim Norway; ^7^ National Marine Fisheries Service, Fisheries Ecology Division Southwest Fisheries Science Center Santa Cruz California USA

**Keywords:** climate change, drought, migration strategy, otolith geochemistry, restoration, salmon

## Abstract

Hydroclimate volatility poses a growing threat to species' persistence, particularly when combined with widespread habitat loss and other stressors. Although the negative impacts of drought on aquatic species are well documented, wet conditions are generally assumed to enhance recruitment of migratory species during freshwater life stages. Life‐history theory predicts that species with multiple migratory strategies should be more resilient to climate extremes by spreading risk across time and space. Testing these assumptions requires tracking both survivors and ‘ghosts’ across life stages and broad spatial scales. For many Pacific salmonids, a substantial fraction of the population disperses downstream soon after emergence (‘early migrants’), rearing in downstream habitats before entering the ocean. The extent to which hydroclimatic conditions influence selection against this phenotype and its lifetime success is poorly understood. To address this data gap, we tracked nine cohorts of Chinook salmon ( *Oncorhynchus tshawytscha* ) at sequential points through their life cycle, using otolith and eye lens isotopes to identify habitats and conditions associated with the highest losses of early migrants. Comparisons of juvenile (*n* = 2197) and adult (*n* = 3937) samples revealed large spatiotemporal variation in the strength of selective mortality. Early migrants became progressively rarer at each consecutive sampling point, including among adult returns, indicating carryover effects from freshwater into the ocean. At the southern edge of the species range—where water scarcity is an intensifying issue—we had predicted positive relationships between river flow, growth and survival. Instead, trends were consistently quadratic, suggesting that both drought and extreme high flows can reduce rearing opportunities and cohort strength. Ultimately, all phenotypes contributed to reproduction in all years, underscoring the importance of maintaining diverse life history portfolios and implementing climate‐ready restoration strategies that provide refugia and growth opportunities across the full spectrum of flow conditions.

## Graphical abstract

We tracked nine cohorts of Chinook salmon ( *Oncorhynchus tshawytscha* ) from California's American River across their life cycle, using otolith and eye lens isotope analysis to identify the habitats and river conditions associated with the greatest losses of early migrants. Return rates, growth in non‐natal habitats, and size‐selective mortality showed consistently quadratic relationships with flow, suggesting that both drought and extreme high flows can limit rearing opportunities and reduce cohort strength. In an increasingly volatile climate, these findings highlight the need for restoration actions that provide refugia and growth opportunities across a broad range of flow conditions.

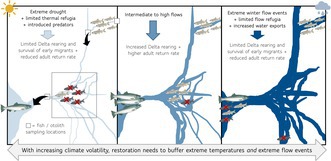

## 1 Introduction

Hydroclimate volatility is having devastating impacts on public health, infrastructure, and food and water security (Swain et al. 2025). In freshwater environments, its negative effects have been amplified by widespread habitat loss and fragmentation related to dam construction and sea level rise, flow alteration, biological invasions, pollution, and warming (Belletti et al. 2020; Fluet‐Chouinard et al. 2023; Tockner and Stanford 2002). Freshwater migratory fishes have fared particularly poorly (Deinet et al. 2020), and billions (USD) are being spent on habitat restoration and protection to reverse declining population trends (Bergstrom and Loomis 2017; Szałkiewicz et al. 2018). However, understanding the efficacy of these programmes is challenging, in part because of the difficulties in understanding the extent and drivers of mortality in aquatic environments (our so‐called ‘ghosts’). Identifying key mortality hotspots and how selective mortality patterns are shaped by hydroclimatic conditions is critical to developing effective and climate‐ready restoration plans (Erwin 2009).

Identifying pressure points requiring management intervention requires understanding how different factors influence different life stages and phenotypes under varying hydroclimatic regimes, and how this translates into population growth or decline. Various approaches have been used to identify survival ‘bottlenecks’ and life stages, locations, and/or stressors that have an outsized influence on population viability (Reed et al. 2002). However, approaches such as population viability analysis have come under criticism (reviewed in Chaudhary and Oli 2020), highlighting the importance of high‐quality empirical data to characterise demographic parameters across life stages. Another challenge to designing effective management measures is that the impacts of stressors may not be realised or detectable until subsequent life stages, or after movement into new habitats, a concept known as ‘carryover effects’ (Gosselin et al. 2018). Although carryover effects can be incorporated into population models, if their dynamics are not well characterised, conclusions may be overgeneralised or inaccurate, resulting in misinformed conservation efforts (O'Connor et al. 2014).

Salmonids exhibit complex life cycles and diverse life histories. When combined with increasingly unpredictable climate conditions, these traits pose significant challenges for management and conservation (Ward et al. 2015; Crozier et al. 2021). Widespread population declines have heightened the urgency to find new ways to improve productivity and resilience (Nicola et al. 2018; Thorstad et al. 2021). Modern management goals explicitly seek to preserve life‐history diversity, as its loss, driven by habitat fragmentation, flow simplification, and hatchery practices, is often cited as a major driver of species decline (Des Roches et al. 2021; Munsch et al. 2022). Here, we explore variation in juvenile outmigration behaviors and how hydroclimatic extremes affect salmon survival in both freshwater and marine environments.

Many salmon populations adopt an ‘early migration’ strategy, where a large fraction of juveniles disperse downstream soon after emergence as ‘fry’. These early migrants typically rear in a series of non‐natal freshwater habitats before entering the ocean months or even years later (Apgar et al. 2021; Koski 2009; Sturrock et al. 2020). Early migration is likely triggered by a combination of storm events and density dependence (Gibeau and Palen 2021; Greene and Beechie 2004; Zeug et al. 2014), but the sheer prevalence of this strategy means that the fate of these individuals—and the quality of the downstream habitats they rely on—can have a disproportionate effect on cohort strength (Sturrock et al. 2020; Apgar et al. 2021). Although multiple factors influence survival in young fish, including migration timing and growth plasticity (Fraser et al. 2007; Cordoleani et al. 2021; Sturrock et al. 2020), selection on size is often a major driver, favoring larger, faster growing individuals (Perez and Munch 2010; Sogard 1997). However, tracking the fate of early migrants remains an ongoing challenge, with their diminutive size and high mortality rates making external tagging impractical. In more modified systems, it has often been assumed that early migrants do not contribute meaningfully to reproduction, but recent studies have shown that large numbers can survive to adulthood and they can dominate the spawning population in some years (Willmes et al. 2024). Overall, however, early migrants do tend to exhibit lower survival rates than late migrants (Sturrock et al. 2020), but without sequential sampling along the migratory corridor, it is impossible to ascertain whether these losses occur primarily in freshwater or the ocean (Cordoleani et al. 2021; Sturrock et al. 2020).

Survivor bias presents intrinsic challenges in aquatic ecology for understanding growth, mortality, and production (Johnson et al. 2014), given that—with the exception of events such as marine mammal strandings and mass fish kills—most animals are simply not observed after death (Ricker 1969). Archival structures provide unique opportunities to ‘go back in time’ and reconstruct the same trait in a group of organisms sampled sequentially through time and/or space to assess the direction and strength of selection over the preceding period and/or area (Woodson et al. 2013). Natural chemical tags recorded in archival structures such as fish otoliths (earstones) and eye lenses provide a valuable tool to reconstruct movement patterns of even the earliest and smallest life stages (Bell‐Tilcock et al. 2021; Sturrock et al. 2015; Willmes et al. 2024). Accordingly, they have been used to infer selection on growth (Woodson et al. 2013) and size (Perez and Munch 2010; Sturrock et al. 2020). Otoliths are calcium carbonate structures located in the inner ear of teleosts that form daily and annual growth layers that incorporate chemical markers that reflect the fish's environmental and physiological chronological experience (Campana 1999). Strontium (Sr) substitutes for calcium in the mineral lattice of otoliths, and because strontium isotope ratios (^87^Sr/^86^Sr) in surface waters vary predictably between different geologic provinces and are recorded in biomineralised tissues without significant fractionation, otolith ^87^Sr/^86^Sr can be a powerful tracer of fish movement (Brennan et al. 2019; Kennedy et al. 1997; Phillis et al. 2018). Eye lenses represent another archival structure that can help to infer fish provenance and movement. They are primarily composed of crystallin protein (Quaeck‐Davies et al. 2018), and the isotopic composition of individual layers (laminae) can be used to infer habitat use and diet (Wallace et al. 2014). In salmon, eye lens stable isotopes can infer whether an individual had been hatchery raised and fed a marine fish meal diet (indicated by elevated δ^34^S and δ^13^C in the laminae deposited immediately post emergence) or born in a river and nourished by freshwater invertebrates (Des Roches et al. 2021).

**FIGURE 1:**
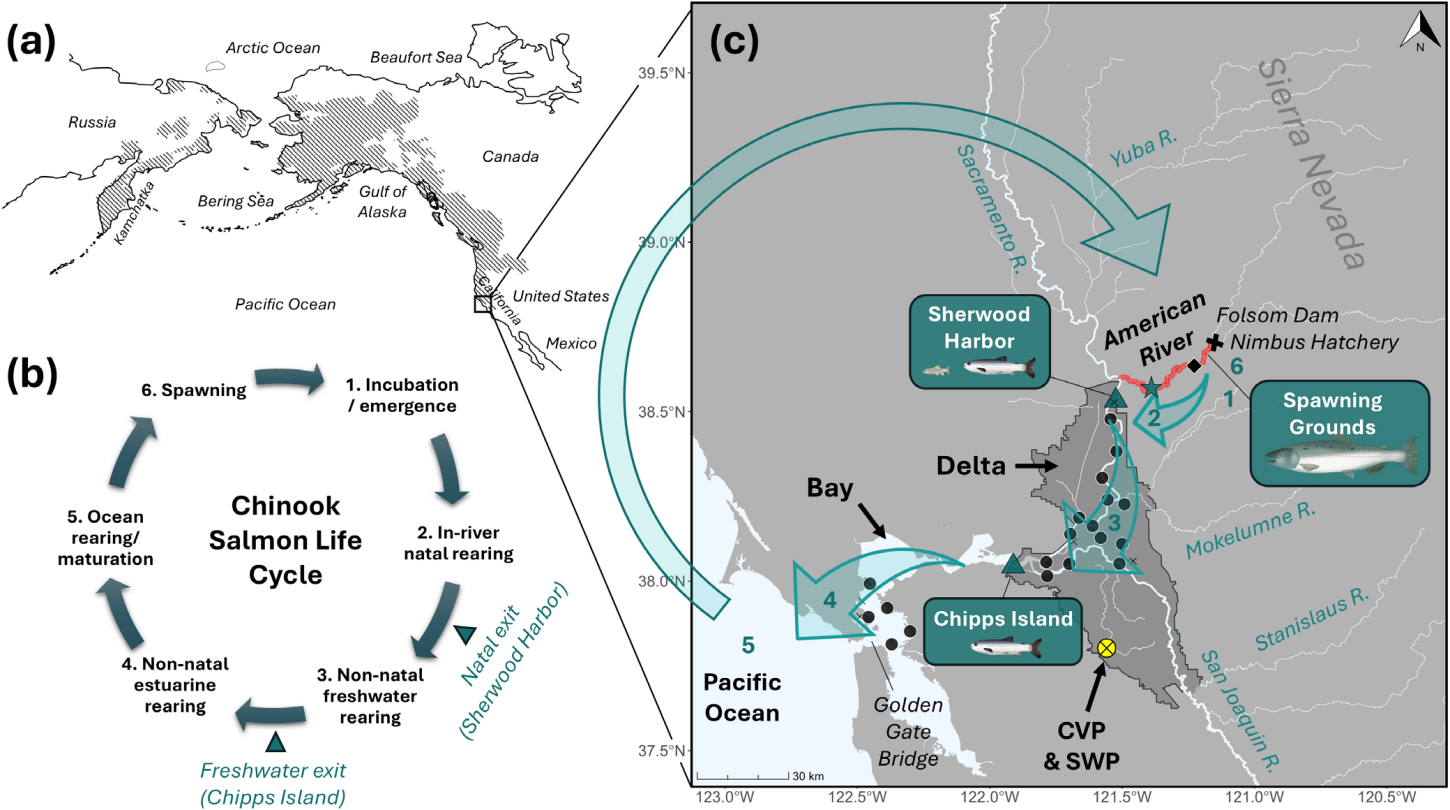
Map showing (a) the Chinook salmon native distribution (hatched area; adapted from Merz et al. 2016), (b) their life cycle with the transition points described in this paper, and (c) the California Central Valley (CCV) with the American River (AME; red) and migration route (green arrows) numbered according to (b). Trawls (green triangles) sampled outmigrating juveniles as they entered and exited the San Joaquin River Delta (legal area indicated by dark grey polygon) at Sherwood Harbor and Chipps Island, respectively. In 2014 and 2015, outmigrating juveniles were also sampled in the rotary screw trap in the lower AME (star). Beach seines also sampled juveniles from sites throughout the Delta and San Francisco Bay (circles and crosses represent sites with vs. without AME origin fish observed). After returning from the ocean 2–4 years later, post‐spawned adult carcasses were sampled from the upper‐AME spawning grounds just below Folsom Dam. The Central Valley Project (CVP) and State Water Project (SWP) water export and fish salvage facilities are indicated by a yellow circle. The USGS gauge at Fair Oaks (11446500), where flow data were collected, is indicated by a diamond, and the location of Folsom Dam, the upstream limit to migration, and Nimbus Hatchery are indicated by a cross. Salmon graphics by Emily Nastase, Claire Sbardella, and Joe Edgerton via the Integration and Application Network (www.ian.umces.edu/media‐library, accessed 19th December 2025).

In the California Central Valley (CCV), dams, habitat loss, droughts, water extraction, flow alteration, bioinvasions, pollution, and other anthropogenic stressors have pushed most Chinook salmon ( *Oncorhynchus tshawytscha* ) populations to the brink of extinction (Katz et al. 2013; Yoshiyama et al. 2001; Munsch et al. 2022). All salmon runs are now dominated or heavily supported by hatchery production (Johnson et al. 2012; Palmer‐Zwahlen et al. 2019), with practices such as trucking smolts directly to the San Francisco Bay (hereon, ‘Bay’) resulting in high levels of straying and genetic and demographic homogenisation (Carlson and Satterthwaite 2011; Sturrock et al. 2019). The Sacramento‐San Joaquin River Delta (hereon, ‘Delta’) represents the only route for juvenile salmon to reach the Pacific Ocean (Figure 1), and one of the most important U.S. water conveyance regions, having been transformed from a vast, rich labyrinth of dendritic rivers and wetlands into a series of engineered, simplified channels (Robinson et al. 2014; Munsch et al. 2022). Early migrants would have historically benefited from the extensive Delta wetlands and floodplains that become reconnected by the same winter storms that displace them downstream (Cordoleani et al. 2022). However, today's Delta may represent an ‘ecological trap’ for early migrants since its relatively recent transformation (Hale and Swearer 2016). Larger smolt life stages moving through the Delta also exhibit some of the lowest survival rates recorded in the system (Buchanan et al. 2018; Michel et al. 2015), particularly in late spring, when higher temperatures and lower water quality result in exceptionally high predation rates (Lehman et al. 2017; Nobriga et al. 2021). Indeed, although ocean conditions are often cited as the main driver of cohort strength in salmonids (Duffy and Beauchamp 2011; Lindley et al. 2009; Thorstad et al. 2021)—analogous to the Atlantic salmon populations struggling in southern Europe (Nicola et al. 2018)—CCV salmon are at the southern edge of the species' range, and their freshwater experience plays a disproportionate role in their survival (Michel 2018; Sturrock et al. 2015, 2020).

The CCV provides a valuable case system to explore the impacts of extreme weather patterns on salmon habitat use and mortality, given its naturally variable Mediterranean climate that has become even more dynamic with climate change, featuring intense droughts punctuated by floods caused by atmospheric rivers (Swain et al. 2025). Previous CCV studies suggest that increased freshwater flows boost juvenile salmon survival and cohort strength (Michel 2018; Notch et al. 2020; Sturrock et al. 2020). However, in other systems, extreme winter flows cause redd (nest) scour and reduced juvenile survival (Ward et al. 2015). Indeed, newly emerged fry tend to be poor swimmers that benefit from low water velocities, high prey densities, and warmer water temperatures (within their physiological tolerance limits) (Sommer et al. 2001). As such, without rearing habitat enhancement using both flow and non‐flow actions such as floodplain reconnection, wood augmentation, increased shade (Sellheim et al. 2016; Yarnell et al. 2015), simply ‘adding more water’ may not have the desired outcome for salmon recovery in this system.

Here, sequential sampling of Chinook salmon from a single population (the American River) was used to track nine cohorts across their lifecycle (outmigrating 2011–2019 and returning to spawn in 2014–2021), to understand how hydroclimatic conditions influence size‐selective mortality patterns along the migratory corridor and through different phases of their lifecycle. As outmigrants exhibit largely unidirectional movement, strategic sampling of juveniles throughout the Delta and Bay, and at habitat transitions characterised by sharp shifts in water chemistry (Barnett‐Johnson et al. 2008) allowed us to track the relative success of different migratory phenotypes through the freshwater Delta (Figure 1). By cohort matching post‐spawned adults in the American River (Figure 1) to the same outmigration years, we explore how juvenile experience and migratory tactics influence lifetime success. On the basis of previous studies (e.g., Sturrock et al. 2020), we hypothesised (1) that we would observe two main migration pulses from the natal river (early and late), and that early migrants would grow in the Delta before leaving freshwater (non‐natal rearing); (2) that size‐selective mortality would result in the prevalence of early migrants decreasing with increasing distance downstream, but that the strength of selection would be higher in the Delta than the Ocean; and finally, (3) that non‐natal rearing in the Delta and cohort replacement rates (i.e., spawner‐to‐spawner return rates) would be positively related to freshwater flows, and that size‐selective mortality would be highest during drought and lowest during wet years.

## 2 Materials and Methods

**FIGURE 2:**
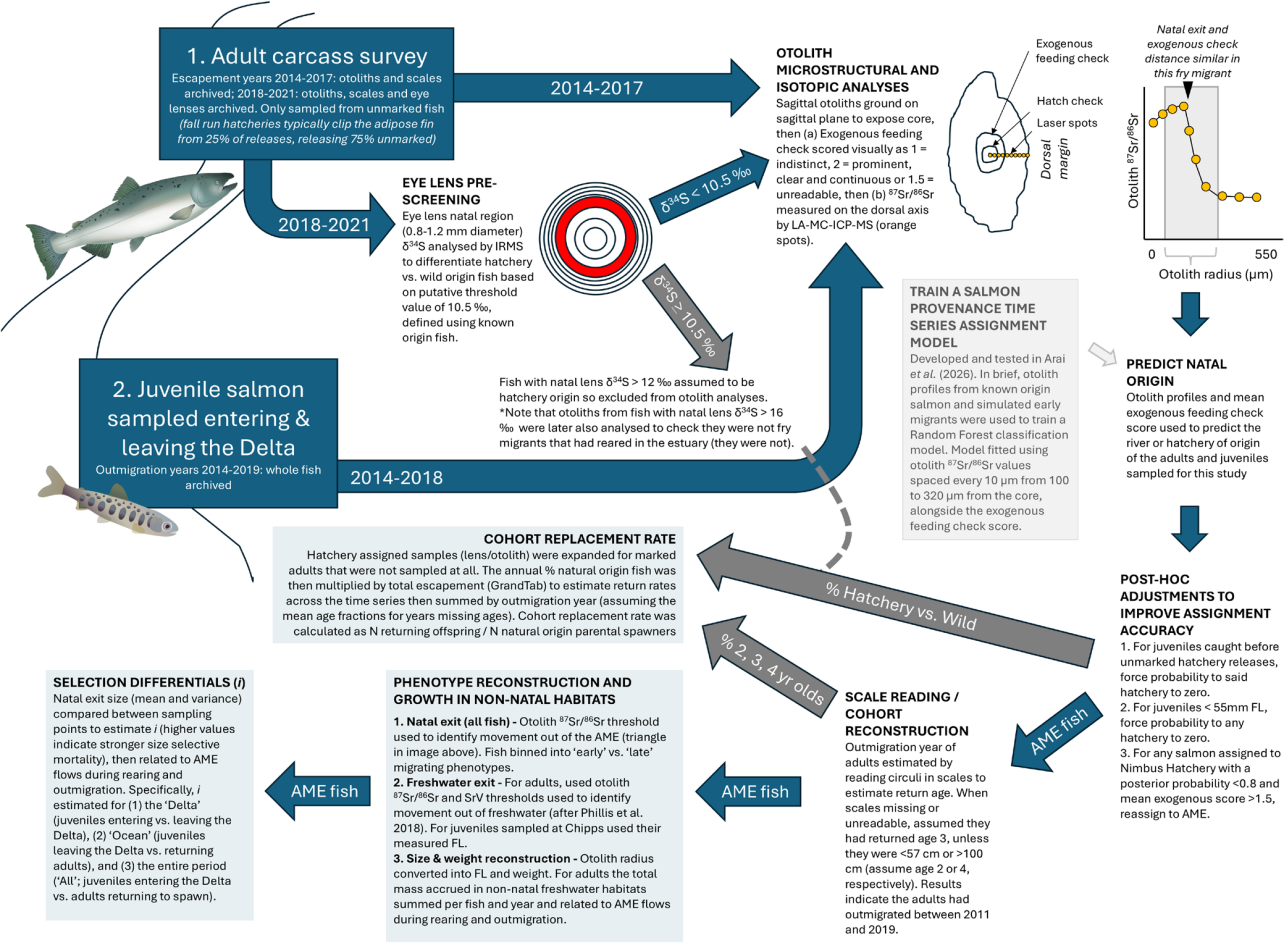
Flow diagram showing all methods used in this paper. Adult and parr salmon graphics by Emily Nastase and Joe Edgerton, respectively, Integration and Application Network (www.ian.umces.edu/media‐library, accessed 19th December 2025).

A flow diagram summarizing all the methods and data used in this study is provided in Figure 2. The underlying code and data are available at https://doi.org/10.6084/m9.figshare.31871866.

### 2.1 Study System and Flow Estimates

The American River (AME) runs from the Sierra Nevada to its confluence with the Sacramento River and the northern boundary of the Delta (Figure 1). Its spawning grounds are situated immediately below Nimbus Dam, the upstream limit to anadromy, where Nimbus Hatchery is also located. In non‐drought years, the region typically experiences increased runoff during October/November, with storms that trigger upstream migration and spawning in adult fall‐run Chinook salmon. Larger storm events come in January and February, coincident with fry emergence from the gravel, followed by spring recession flows in April and May as snowpack melts. Nearly all fall‐run CCV Chinook salmon outmigrate to the ocean as subyearlings within 6 months of emergence, leaving freshwater past Chipps Island (Figure 1), typically from April to June. Mean daily flows in the AME at Fair Oaks (USGS gauge number 11446500, Figure 1) were obtained from https://waterdata.usgs.gov (accessed 11 August 2024). Mean daily Delta outflows (‘Dayflow’) were obtained from https://water.ca.gov/ (accessed 10 October 2024). Throughout, we refer to flows in the AME, but we are confident that they provide a good proxy for system‐wide runoff, being highly correlated with Delta outflows (*F*_1,7_ = 264.3, *p* < 0.0001, adj. *r* ^2^ = 0.97 using logged mean Jan‐June flows).

### 2.2 Sample Collections

#### 2.2.1 Juvenile Sampling

In 2014–2019 the Delta Juvenile Fish Monitoring Program (DJFMP) lethally subsampled unmarked juvenile salmon from their catches under scientific collection permit SC‐12811 (Figure 1). Samples were generally collected across the full January to June outmigration period and randomly selected from within the ‘fall run’ size range indicated by Length at Date Fisher Tables (≤ 2 to ≤ 15 individuals per day, depending on site and year), resulting in a total sample size of 2197 (Table S1). The sites selected provided samples of juveniles (a) entering the Delta by Kodiak Trawl at Sherwood Harbor, immediately downstream of the mouth of the AME, (b) leaving the Delta by midwater trawl at Chipps Island, which is typically coincident with the 0–2 PSU halocline, and (c) throughout the Delta and Bay, sampled by beach seine (15.2 m × 1.3 m, 0.3 cm^2^ mesh) (Figure 1). Juvenile migrants were also sampled (non‐lethally) in the lower AME by rotary screw trap (RST) (Figure 1). Daily passage was estimated by the CAMP Rotary Screw Trap Platform (code and methods provided in https://github.com/tmcd82070/CAMP_RST), and in 2014 and 2015, the measured fork lengths (FL) of unmarked fish (expanded for daily passage) were used to infer the size distribution of AME‐origin fish entering the Delta.

#### 2.2.2 Adult Sampling

Given their semelparous life history, post‐spawned Pacific salmon represent a rare opportunity to sample large numbers of fish posthumously. Here, salmon carcasses (*n* = 3937) were sampled from the AME spawning grounds as part of the CDFW annual carcass survey in 2014–2021 (Table 1, Figure 1). Hatchery origin carcasses (indicated by a clipped adipose fin—typically applied to 25% of hatchery releases) were not sampled, as the study objective was to reconstruct juvenile behaviors of fish that had experienced natural river rearing conditions as juveniles. Each fish had its FL, sex, and sampling location recorded, and both otoliths were extracted and stored dry in 1.5 mL Eppendorf tubes. From 2018 to 2021, eyes were also extracted and stored frozen for future lens extraction. If the carcass was not too degraded, scales were also taken from the dorsal flank of the fish and stored dry in coin envelopes for age analysis.

**TABLE 1:** Adult carcass sampling dates and sample sizes for otolith and eye lens chemistry analyses and scale reads, the fractions and numbers assigned to the American River (AME), and the return age fractions among scale‐read AME‐assigned individuals.

Escapement year	Carcass sampling date range		Total *N* fish analyzed	*N* prescreened for hatchery/wild origin using eye lens δ^34^S	*N* useable otolith ^87^Sr/^86^Sr profiles	% of fish assigned to AME (note: of unmarked fish)	*N* assigned to AME	% AME assigned fish with assumed age	AME % 2 year old returns	AME % 3 year old returns	AME % 4 year old returns
2014	03/11/14	22/12/14	799	0	790	47%	370	64%	6%	45%	48%
2015	13/11/15	04/01/16	804	0	788	46%	359	45%	10%	75%	16%
2016	31/10/16	04/01/17	250	0	246	39%	95	60%	11%	68%	21%
2017	06/11/17	17/01/18	798	0	792	11%	91	89%	10%	70%	20%
2018	29/10/18	27/12/18	365	365	25	6%	22	14%	26%	68%	5%
2019	04/11/19	31/12/19	301	301	54	13%	46	35%	20%	77%	3%
2020	02/11/20	19/01/21	333	333	42	10%	39	28%	7%	43%	50%
2021	01/11/21	29/12/21	286	285	49	13%	43	26%	3%	88%	9%
Total			3937	1284	2786		1065				

*Note:* When readable scales were not retrieved, the individual was assumed to be 3 years old unless they had an FL < 57 cm (assumed 2) or > 100 cm (assumed 4). Some otoliths were good enough quality to use for natal assignment but became vateritic before natal exit; thus, sample sizes may differ slightly among tables.

### 2.3 Cohort Reconstruction of Adult Fish

Scale preparation and ageing followed the general methods described in MacLellan and Gillespie (2015). The three most legible scales from each sample were mounted between two microscope slides for imaging. Scales were imaged using a Motic BA310 compound microscope with a Motic Cam 5+ camera, and measured at 40× magnification using Image Pro Premier version 9.2 software (Media Cybernetics, Rockville, Maryland). Scales were independently read by two readers and resolved by a more senior reader if a difference was found (12% of samples). If scales were unreadable, the fish was assumed to have returned age‐3, given that this is the dominant return age for natural origin salmon in this system (74%–88% of natural origin fish from years with > 5 samples from Willmes et al. 2024 and Table 1). However, if their FL was < 57 cm or > 100 cm, they were reassigned as 2 or 4 (*n* = 18 fish), respectively, on the basis of apparent breakpoints in the FL size distribution (Figure S1) and conservative size thresholds of known‐age 2‐ and 4‐year‐old fish (Morais et al., in review).

### 2.4 Natal Assignments

#### 2.4.1 Otolith Chemistry and Microstructural Analyses

Sagittal otoliths were ground and polished to the core on the sagittal plane using methods described in Sturrock et al. (2015). The prominence of the exogenous feeding check was scored as 1 (indistinct), 2 (sharp and distinct all the way round the core), or 1.5 (not possible to score) after Barnett‐Johnson et al. (2007). This microstructural check mark was used to support natal assignments, on the basis of the assumption that wild fish experience increased stress during the period immediately post‐emergence because of lack of food, leading to the formation of a distinct exogenous feeding check. We then measured strontium isotope ratios (^87^Sr/^86^Sr) following established protocols (Sturrock et al. 2015) using a Nd:YAG 213 nm laser (New Wave Research UP213) coupled to a Nu Plasma HR MC‐ICP‐MS (Nu032) at the University of California, Davis Interdisciplinary Center for Plasma Mass Spectrometry (Table S2). All transects followed the same standardised axis on the dorsal plane from the core to the otolith edge (juveniles), or from the core to an otolith radius of *c*.1000 μm in adults, once ^87^Sr/^86^Sr values reached ~0.70918 and increasing SrV values had indicated outmigration to the ocean.

#### 2.4.2 Eye Lens Isotope Analyses

Because of the high numbers of unmarked hatchery origin fish (that were decoupled from river conditions experienced by natural origin juveniles), carcasses sampled from 2018 to 2021 were ‘pre‐screened’ for hatchery vs. wild origin using eye lens sulfur signatures (δ^34^S) in the natal region of the eye lens (lens diameter = 800–1200 μm; with Bell‐Tilcock et al. 2021). With using the cutoff inferred by known‐origin hatchery vs. wild salmon (see Text S1), individuals with lens δ^34^S values ≤ 10.5‰ were selected as potentially natural origin fish, and their otoliths were analysed for ^87^Sr/^86^Sr using the methods outlined above. In addition, we also analysed otoliths from fish with lens natal δ^34^S values > 16‰ (Figure S2) to check that these individuals were not early migrants that had been displaced into the Bay and reared in brackish water.

#### 2.4.3 Natal Assignment Model

Juvenile and adult samples were assigned to a natal river or hatchery using a random forest (RF) model implemented in the R package *ranger* (Wright and Ziegler 2017). Full details are provided in Arai et al. (2026), but in brief, we used the ‘discrete time series’ model, trained on sequential otolith ^87^Sr/^86^Sr isotope measurements from an otolith radius of 100 to 320 μm, alongside the mean exogenous feeding check score. Training data (*n* = 255) included otolith ^87^Sr/^86^Sr profiles from known‐origin juveniles sampled from their natal river or hatchery before outmigration and simulated profiles of early migrants that reared in the Delta (Arai et al. 2026). Inclusion of simulated profiles in the training data improved identification of presumed AME‐origin early migrants from 75% to 100% accuracy (Arai et al. 2026). Overall, Arai et al. (2026) reported a cross‐validated assignment accuracy for AME‐origin fish of 60.7% ± 2.1% (mean ± SD), with all misclassifications being with Nimbus Hatchery (NIH) on the same river (NIH accuracy = 86.7% ± 0.0%). Applying the model to a fully independent dataset of known‐origin fish indicated higher accuracy when early migrants comprised a larger fraction of the test dataset (81% accuracy with 48% early migrants vs. 63% accuracy with zero early migrants).

To improve accuracy, two *post hoc* reassignment steps were performed. First, we used the mean exogenous feeding check score, supported by observed differences between known‐origin AME smolts (1.61 ± 0.36 SD) *cf*. NIH smolts (1.16 ± 0.21). Using our independent test dataset (*n* = 16 AME and *n* = 18 NIH smolts), we evaluated scenarios reassigning NIH‐assigned fish with differing class probabilities (< 0.6, 0.7, 0.8, or 0.9) and exogenous feeding check scores > 1.5 to AME, and vice versa for reassigning AME‐assigned fish to NIH. All scenarios improved assignment accuracy (Figure S3), but reassigning AME‐assigned fish to NIH resulted in substantial misclassification of known‐origin AME fish to NIH (25%–31%). We therefore adopted the scenario reassigning only NIH‐assigned fish with class probabilities < 0.8 and exogenous scores > 1.5 to AME, yielding 81% AME and 94% NIH accuracy. When applied to unknown‐origin adults, this resulted in the reassignment of 4.3% of individuals (Figure S4).

The second set of reassignment steps was applied to juveniles only (in addition to step 1). Hatcheries did not release fry during the study period, so juveniles < 55 mm FL were assumed natural origin, and juveniles sampled before a hatchery's first release of unmarked fish in a given year were presumed not to originate from that hatchery. We also assumed that juveniles entering the Delta from southern tributaries (Stanislaus, Tuolumne, Merced Rivers) would not migrate upstream to the Sherwood Harbor trawl site. In each case, the corresponding class probability was forced to zero, and the fish was reassigned to the source with the next highest probability. Collectively, these steps resulted in 13% of juveniles being reassigned, primarily on the basis of sampling date preceding the first hatchery releases date, highlighting the difficulty of determining provenance of small fish and the value of additional markers such as eye lens δ^34^S.

### 2.5 Outmigration Size and Mass Reconstructions

#### 2.5.1 Natal Exit

**FIGURE 3:**
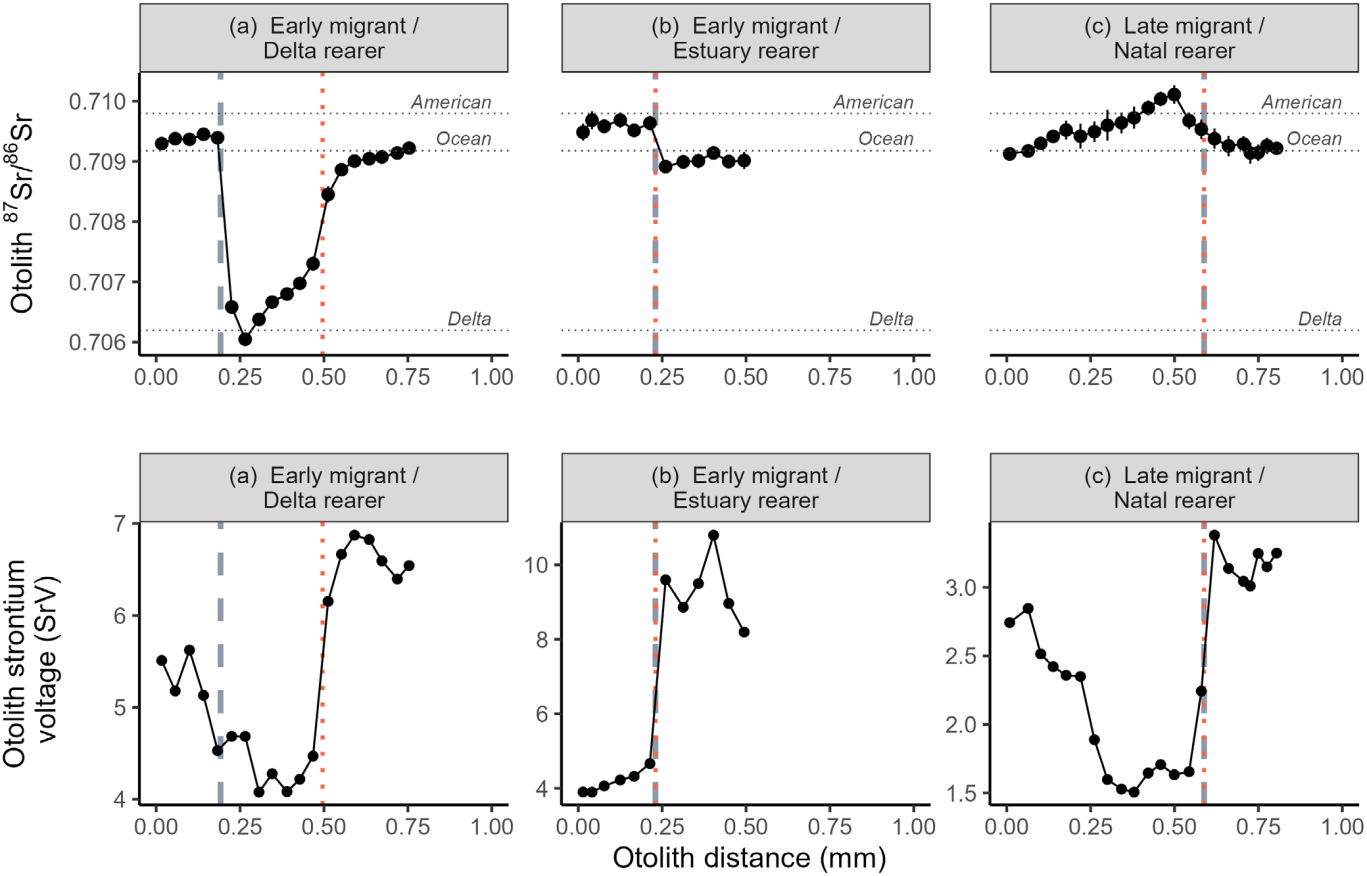
Paired otolith ^87^Sr/^86^Sr (± 2SE; top row) and strontium concentrations (SrV; bottom row) profiles from three example salmon, sampled as returning adults (a and c) or as a juvenile in the Bay (b). Plot (a) shows a typical early migrant that reared in the Delta before going to sea, where otolith ^87^Sr/^86^Sr thresholds were used to estimate natal (grey dashed line) and freshwater (red dotted vertical line) exit size. (b) The only fish in the study that spent a detectable period rearing in brackish water in the Bay. Plot (c) A late migrant that reared only in the natal river before migrating rapidly to the ocean. As otolith ^87^Sr/^86^Sr profiles for (b) and (c) did not cross the AME or Delta thresholds because of rapid movement through the Delta, otolith SrV (normalised within fish, see Methods) was used to estimate freshwater exit. Natal exit was then matched to this, assuming zero Delta growth. Mean AME, Delta, and Ocean ^87^Sr/^86^Sr values are indicated by horizontal lines.

The transition from the AME to the Sacramento River (northern Delta) was termed ‘natal exit’ and identified on the basis of changes in otolith ^87^Sr/^86^Sr measurements along the dorsal axis. Specifically, natal exit was identified as the first departure from the range of values observed in the American River (i.e., using ‘threshold analysis’, after Cordoleani et al. 2021; Sturrock et al. 2015, 2020; Willmes et al. 2024). Given that the AME has ^87^Sr/^86^Sr values higher than all other salmon rivers in the CCV, the minimum water ^87^Sr/^86^Sr value observed in the AME (0.7085) practically served as the natal exit threshold, using linear interpolation between neighboring measurements to estimate the otolith radius at which the profile crossed this value (Figure 3a). Some otolith ^87^Sr/^86^Sr profiles remained above this threshold for their entirety, either because the fish had only just left the natal river or had traveled to sea so rapidly that their otolith did not register the Delta's isotopic decrease (e.g., Figure 3b,c). When this occurred in juveniles caught upstream of Chipps Island, we used their total otolith radius as their natal exit size, and when observed in adults or Bay‐sampled juveniles, we used their freshwater exit size (inferred by SrV, see below) as their natal exit size. For juveniles caught in the RST, we used their measured FL as their natal exit size.

#### 2.5.2 Freshwater Exit

For juveniles sampled leaving the Delta at Chipps Island, measured FL was interpreted as their freshwater exit size. For Bay‐sampled juveniles and returning adults, freshwater exit size was estimated using linear interpolation to estimate the first point after natal exit that the profile crossed the mean ^87^Sr/^86^Sr value at Chipps Island (0.70785), after Phillis et al. (2018). For individuals where this threshold was not crossed (e.g., Figure 3B,C), relative otolith SrV was used as a proxy for strontium concentrations, updating the threshold value from 66% (Phillis et al. 2018) to 52% as this reduced the within‐fish difference in freshwater exit sizes estimated using ^87^Sr/^86^Sr and SrV.

#### 2.5.3 Migratory Phenotype Assignment

Hartigan's dip test (Hartigan and Hartigan 1985) was used to test whether exit sizes were unimodal. Bimodality was observed for natal exit sizes, with the valley occurring at a smaller size than the 55 mm FL (specifically, an otolith radius cutoff of 398 μm) used in previous studies (Sturrock et al. 2015; Willmes et al. 2024). As such, mixed distributions were fitted using the *normalmixEM* function in the *mixedtools* R package (Young et al. 2006), and the *uniroot* function was used to find the otolith radius where the difference between the two density functions equalled zero (Figure S5A). ‘Early’ and ‘late migrants’ were then assigned if they had left the natal river smaller or larger than this value, respectively.

#### 2.5.4 Length and Mass Reconstruction

Otolith radius from the core along the dorsal axis was converted into FL using a broken stick linear regression in the *segmented* package. The relationship (Equation 1) was fitted using juveniles of known otolith radius and FL, using the individuals from Sturrock et al. (2020) updated with additional fall run juveniles to improve accuracy (*n* = 813, adj. *r* ^2^ = 0.88; Figure S6A). To estimate the mass assimilated in natal and non‐natal freshwater habitats, otolith radius was converted into mass using Equation (2) that was fitted using known‐size fall run juveniles (*n* = 481, adj. *r* ^2^ = 0.91; Figure S6B). The change in mass between emergence (assumed size of 30 mm or an otolith radius of 215 μm) and natal exit, and between natal exit and freshwater exit was then used to estimate the mass in grams accumulated in natal vs. non‐natal regions, then normalised to total freshwater growth for that individual to compare more easily among fish (Brennan et al. 2019).

(1) $$\mathrm{FL}\;\mathrm{in}\;\mathrm{mm}\;=\;\left\{\begin{array}{ll} 0.021259\cdot \mathrm{OR}+31.08748, & \mathrm{if}\;\mathrm{OR}<256\\ 0.165660\cdot \mathrm{OR}‐5.8799, & \mathrm{if}\;\mathrm{OR}\geq 256 \end{array}\right.$$

(2)

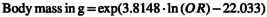

where *OR* is the otolith radius from the core towards the dorsal edge in microns.

### 2.6 Estimating Selection Differentials

To estimate selection on size (*i*), natal exit sizes were compared between AME‐origin fish sampled at earlier vs. later time points using Equation (3), after Perez and Munch (2010) and Falconer and Mackay (1996). Specifically, *i* was estimated annually for the ‘Delta’ (juveniles entering vs. leaving the Delta, 2014–2018) and ‘Ocean’ (juveniles leaving the Delta vs. adults spawning 2–4 years later; 2014–2019). Given low sample sizes for Chipps Island in some years, we also estimated *i* for the entire period after leaving the natal river, on the basis of juveniles entering the Delta vs. adults returning to spawn (‘All’, 2014–2018). To explore drivers of selection, *i* values were then regressed using linear and quadratic models against mean natal river flow, the mean size of migrants at Delta entry (the earliest sampling point), and total escapement the previous fall as a proxy for juvenile densities.

(3) $$i=\frac{{{\bar{z}}^{*}}_{t}-{\bar{z}}_{t}}{\sqrt{v_{t}}}$$

where ${{\bar{z}}^{*}}_{t}$ is the mean FL at time t of the surviving population, ${\bar{z}}_{t}$ is the mean FL at time t in the initial population, and $v_{t}$ is the variance in length at time t in the initial population.

As there were insufficient or temporally imbalanced samples collected from the Sherwood Harbor trawl in 2014 and 2015 (Figure S7), we used the size distribution observed in the lower AME RST to represent ‘Delta entry’ for these two cohorts, using a random sample of 100 fish per year to roughly match sample sizes among methods. Unlike the other years in the time series, 2014 and 2015 had no in‐river hatchery releases upstream of the RST, so we could be confident that all samples were AME‐origin, and the traps were consistently operated through the season (Figure S5C). However, the RST size distribution could be skewed smaller than the true natal exit size distribution, given its location 9 river miles upstream of the confluence and the isotopic breakpoint used to estimate natal exit (Figure 1). As such, we performed a sensitivity analysis where we expanded otolith samples from trawl samples to match the observed catch distributions using a range of different time binning scenarios (Text S1). The results were broadly similar if we used the expanded catch at Sherwood Harbor data or the RST data (Figure S8), so we used the RST data for 2014 and 2015, given better data availability and through‐season sampling (Figures S6 and S7).

### 2.7 Estimating Cohort Replacement Rate

To assess whether overall survival is associated with freshwater flows experienced during rearing and outmigration, we related the cohort replacement rate of natural‐origin fish to mean Jan‐June AME river flows. To estimate the total numbers of hatchery and natural origin spawners, we multiplied the number of hatchery‐assigned samples by 1.33 (1/0.75) to correct for the marked (clipped adipose fin) hatchery fish that we did not sample otoliths from, representing 25% of hatchery releases. We then calculated the true fractions of wild and hatchery fish using this expanded hatchery count and multiplied these fractions by the total escapement obtained from CDFW GrandTab (Azat 2023). Final estimated abundance of hatchery fish did not differ from those generated by the Constant Fractional Marking (CFM) Program for matching years (Figure S9A; *t* = −0.655, df = 6, *p* = 0.537), so we used CFM estimates for years without otolith data to estimate cohort replacement rates. For escapement years missing age estimates (2010–2013; 2022), we applied the mean age fractions from other years (Table 1), and to estimate the number of age‐4 spawners in 2022 returning from outmigration year 2019, we applied the same hatchery fraction as the year prior (0.87; Table S5), as we did not have otolith‐ or CFM‐derived estimates for that year. We also repeated the analysis assuming that all fish returned age‐3 (i.e., avoiding the age‐ and hatchery‐fraction assumptions mentioned above), and the result was very similar to one reported in this paper (Figure S9B).

## 3 Results

### 3.1 Variation in Hydrology and Outmigration Size

**FIGURE 4:**
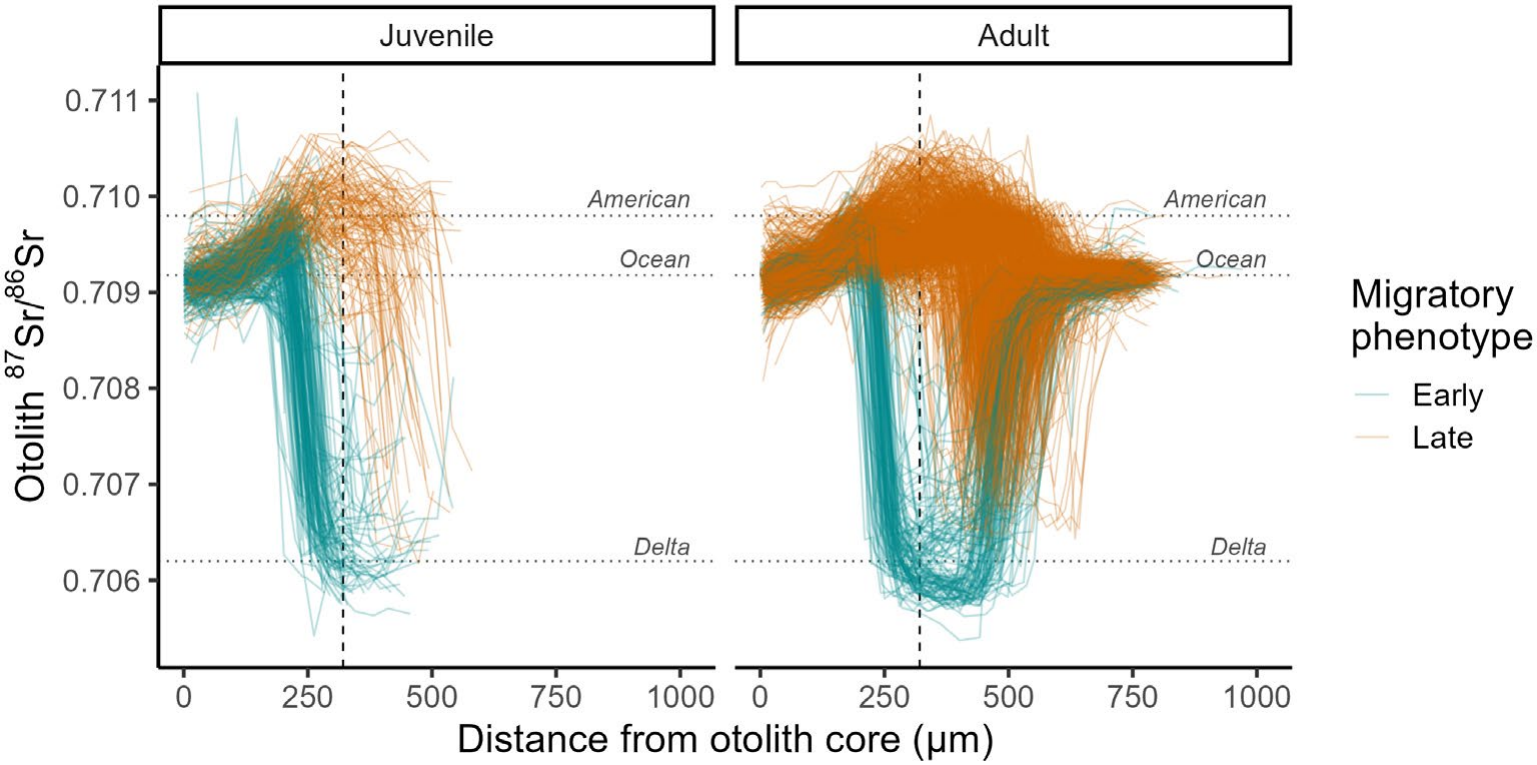
Otolith strontium isotope profiles for all AME‐assigned salmon showing chronological measurements from birth (core) to when they were caught (juveniles; cohorts 2014–2019) or had left freshwater (adults; cohorts 2011–2019). Profiles are colored by migratory phenotype (i.e., “early” vs. “late”, inferred by isotopic departure from the natal river range at an otolith radius equivalent to a fork length ≤ or > 47 mm, respectively, shown by dashed line). The mean isotopic values for the AME, freshwater Delta, and ocean are indicated by horizontal reference lines.

**FIGURE 5:**
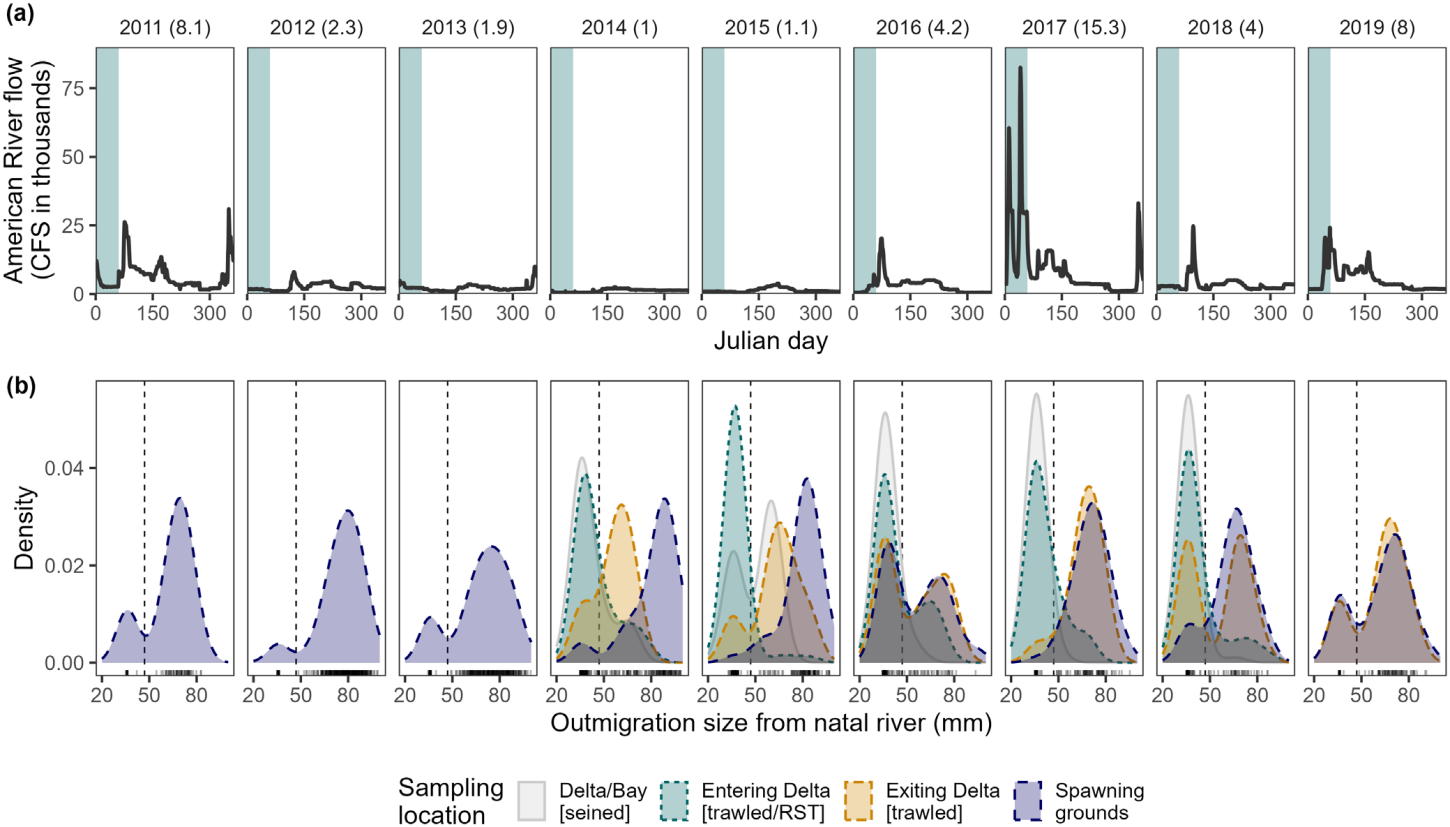
(a) The flows experienced by fish in this study in their natal river (AME), with the main migratory period for early migration (Jan‐Feb) highlighted in blue and the mean AME flow across the migratory period (Jan‐June) in CFS × 10^−3^ in parentheses. (b) Density plots showing reconstructed natal exit size of adult spawners, separated by outmigration year (2011–2019), with raw size data shown as tick marks. The threshold used to identify early migrants (47 mm) is indicated by a vertical dashed line. Colors indicate sampling location, with juveniles collected throughout the Delta and Bay (gray), as they entered (teal) and exited (orange) the Delta, and post‐spawned adults sampled in the AME (blue). Given that beach seines (gray) select for fish actively rearing along river margins and catch fewer fish migrating in the main channel, they are shown simply to demonstrate the prevalence of early migrants through the system in all years. The Delta entry sample (teal) was collected by rotary screw trap in the lower AME in 2014–15 and by Kodiak trawl just downstream of the AME mouth in 2016–18 (Figure 1).

AME‐origin fish exhibited two main migration pulses out of the natal river ('early' and 'late'), coincident with winter storm events in January and February, and then snow melt and warming in April to May (Figure 4, Figure 5). They also experienced a wide range of river flows through the time series, including extremely dry conditions during drought years 2014–2015 and extreme high flows during the wet year 2017, particularly during the peak early migration period (Figure 5A).

**FIGURE 6:**
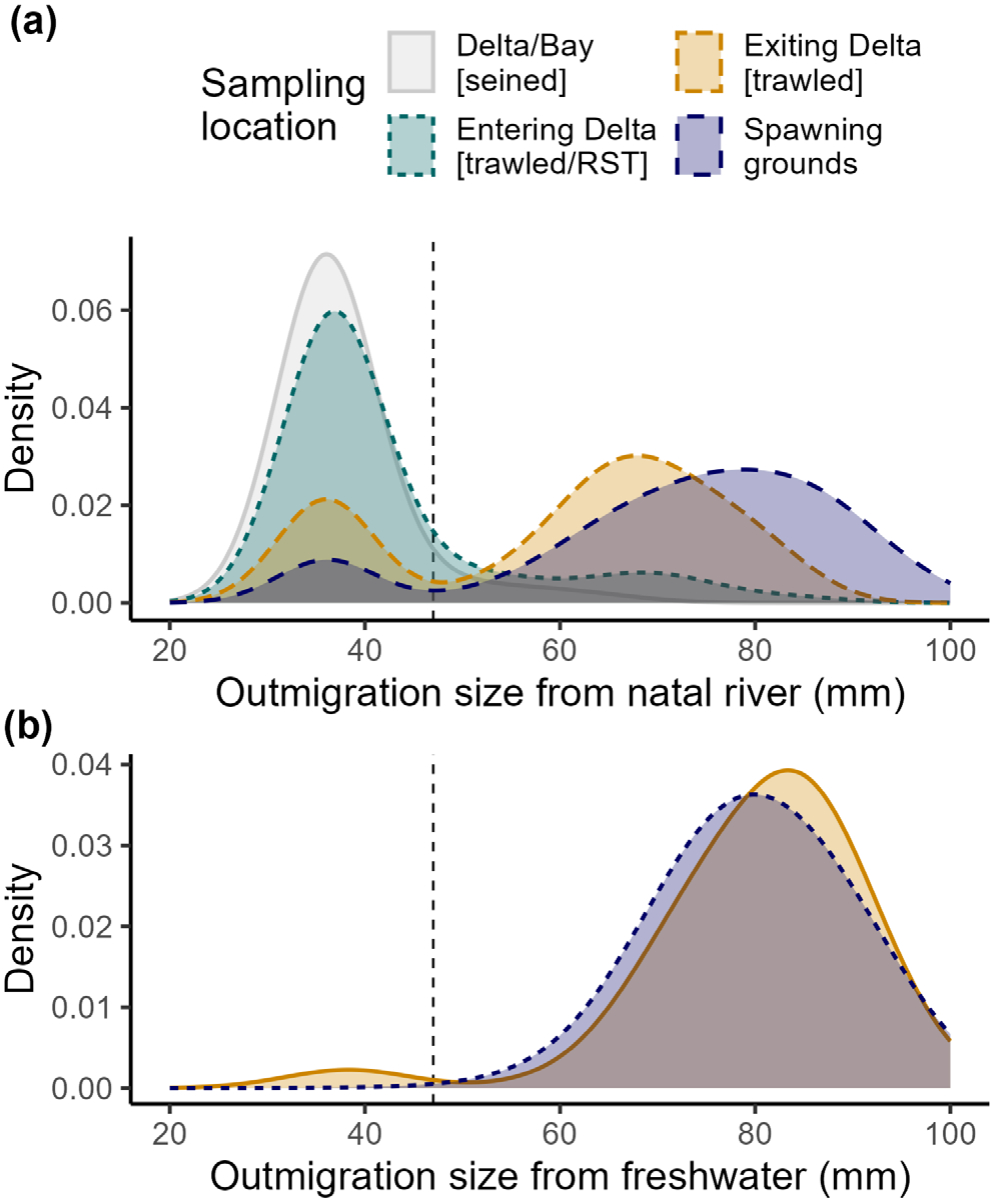
Density plots showing reconstructed (a) natal exit size vs. (b) freshwater exit size for all cohorts and samples combined. Freshwater exit size on the basis of measured fork length of juveniles sampled at Chipps Island (2014–19) and reconstructed size in post‐spawned adults (outmigration years 2011–19). Colors indicate sampling location, with juveniles collected throughout the Delta and Bay (gray), as they entered (teal) and exited (orange) the Delta, and post‐spawned adults sampled in the AME (blue). The threshold distinguishing early migrants (47 mm) is indicated by a vertical dashed line. The Delta entry sample (teal) was collected by rotary screw trap in the lower AME in 2014–15 and by Kodiak trawl just downstream of the AME mouth in 2016–18 (Figure 1).

There was strong evidence to support multimodality in natal exit sizes (Hartigan's dip test, *D* = 0.086, *p* < 0.0001), with ‘early migrants’ leaving the AME as newly emerged fry, typically at 30–45 mm FL and late migrants leaving the natal river over a broader time and size range, but typically at 60–85 mm (Figure 5, Figure 6A). The threshold identified to define an individual as an early migrant was ≤ 321 μm otolith radius, equivalent to ≤ 47 mm FL, supported by the size distributions observed at the lower river RST (PSMFC 2014) (Figure S5). Note that this is smaller than the threshold used in previous studies (55 mm FL used in Miller et al. 2010; Sturrock et al. 2015, 2020; Willmes et al. 2024), so summary data in Table 2 are reported using both thresholds to facilitate cross‐study comparisons.

**TABLE 2:** Sample sizes of AME origin salmon and the fork length (FL) at which they left the natal river and freshwater.

Sample collection region	Outmigration year	Mean Jan‐June flow in the natal river (CFS)	*N* salmon assigned to AME	Natal exit size (mm)		Percentage of early migrants		Freshwater exit size (mm)	
				Mean	SD	< 47 mm cutoff	< 55 mm cutoff	Mean	SD
Entering Delta (RST/KDT)	2014 [RST]	981	100	45.0	12.4	73.0	85.0		
	2015 [RST]	1133	100	38.9	8.6	95.0	95.0		
	2016 [KDT]	4240	28	44.6	13.5	67.9	78.6		
	2017 [KDT]	15,264	10	41.6	10.7	70.0	90.0		
	2018 [KDT]	3998	40	42.7	13.6	80.0	87.5		
	All years			42.6	2.1	77.2	87.2		
Throughout Delta and Bay (beach seine)	2014	981	7	41.9	11.6	71.4	85.7		
	2015	1133	5	50.4	13.2	40.0	80.0		
	2016	4240	105	37.6	4.9	93.3	100.0		
	2017	15,264	38	36.4	2.4	97.4	100.0		
	2018	3998	54	36.9	4.3	98.1	98.1		
	All years			40.6	4.8	80.1	92.8		
Exiting Delta (MWT)	2014	981	5	56.0	12.9	20.0	60.0	73.2	2.2
	2015	1133	6	63.5	16.3	16.7	33.3	73.3	17.5
	2016	4240	26	53.9	18.0	46.2	57.7	81.3	7.9
	2017	15,264	15	66.4	11.2	6.7	13.3	84.5	6.4
	2018	3998	9	55.3	18.6	44.4	44.4	78.9	5.6
	2019	7977	50	62.3	15.7	22.0	26.0	79.4	13.2
	All years			59.6	2.9	26.0	39.1	78.4	5.6
Spawning grounds (Carcass survey)	2011	8053	67	62.8	14.3	19.4	23.9	75.0	6.7
	2012	2276	319	75.7	14.1	6.6	9.7	81.8	8.9
	2013	1900	311	68.9	17.6	16.1	23.2	78.6	9.1
	2014	981	115	81.4	16.1	7.0	10.4	85.6	8.6
	2015	1133	89	79.4	12.2	3.4	10.1	81.9	8.5
	2016	4240	21	55.5	18.3	47.6	52.4	77.7	9.2
	2017	15,264	58	70.5	10.9	1.7	13.8	76.5	9.3
	2018	3998	31	63.7	13.6	12.9	25.8	75.8	7.2
	2019	7977	42	62.5	17.0	23.8	33.3	76.9	9.1
	All years			68.9	2.5	15.4	22.5	78.9	0.9

*Note:* Summary data are reported by collection region, with juveniles sampled by rotary screw trap (RST) in the lower AME, the Sherwood Harbor Kodiak Trawl (KDT), beach seines, and the Chipps Island Midwater Trawl (MWT), and post‐spawned adults sampled on carcass surveys just below Folsom Dam in the American River (Figure 1). We report the percentage that left the natal river early, on the basis of the 47 mm FL cutoff used in this study and the 55 mm FL (specifically, an otolith radius of 398 μm) cutoff used in Sturrock et al. (2015, 2020) and Willmes et al. (2024).

Unlike natal exit sizes, ocean entry sizes exhibited a unimodal distribution (Hartigan's dip test, *D* = 0.008, *p* = 0.91; Figure 6B), centered around a mean FL of 80.1 ± 9.38 mm SD, with most early migrants reaching this size through Delta rearing (Figures 3 and 4). There was no difference in freshwater exit size between juveniles sampled as they left freshwater at Chipps Island (i.e., measured FL) vs. adult returns (i.e., reconstructed FL; *t*_126.6_ = −0.19, *p* = 0.85), but early migrants entered the Ocean significantly smaller than late migrants (means = 73.4 ± 9.98 mm SD vs. 81.1 ± 8.88 mm, respectively; *t*_185.6_ = −8.84, *p*‐value < 0.0001).

### 3.2 The Ghosts: Size‐Selective Mortality Between Sampling Points

**FIGURE 7:**
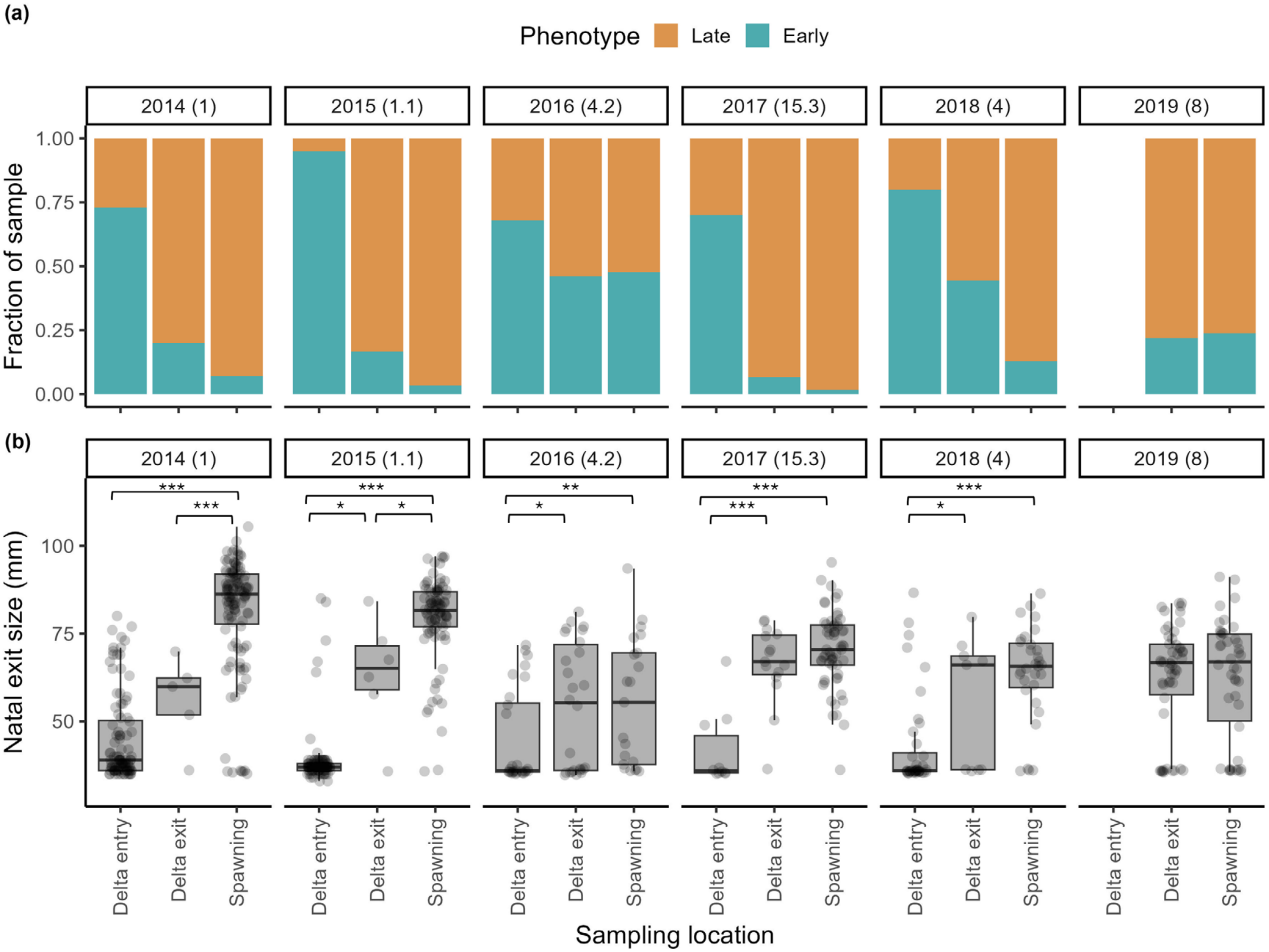
(a) Fraction of early (teal) and late (orange) migrants and (b) median size at natal exit in salmon collected as juveniles entering vs. leaving the Delta vs. returning to spawn 2–4 years later, with significant differences in size between sampling points indicated by asterisks (Tukey or Holm‐corrected Dunn pairwise comparisons: ***p < 0.001, **p = 0.01, *p < 0.08). Mean January to June flow in the AME in CFS × 10^−3^ shown in parentheses. The Delta entry sample was collected by rotary screw trap in the lower AME in 2014–15 and by Kodiak trawl just downstream of the AME mouth in 2016–18 (Figure 1).

The frequency of early and late migrating phenotypes varied among sampling points and years. Overall, the fraction of early migrants at each sampling point ranged from 1.7% in adult returns from outmigration year 2017 to ≥ 97% in juveniles sampled within the Delta in 2017 and 2018 (Table 2). The mean natal exit size and fraction of late migrants (*cf*. early migrants) consistently increased with time and distance from the natal stream, indicating size‐selective mortality through the Delta that often carried over into the ocean (Figures 6, 7). Early migrants represented, on average, 80% of juveniles entering and residing within the Delta, 26% leaving it, and only 15% returning as adults (Table 2). In all years with paired samples, the natal exit size of returning adults was significantly larger than for juveniles sampled as they entered the Delta (Figure 7). Differences in natal exit size were also observed between fish sampled entering vs. leaving the Delta in 2015, 2016, 2017, and 2018 (Tukey or Holm‐corrected Dunn Tests, *p* < 0.08), and also between fish leaving the Delta in 2014 and 2015 vs. returning to spawn (Tukey or Holm‐corrected Dunn Tests, *p* < 0.07), median natal exit size was nearly identical between fish leaving the Delta vs. returning to spawn in 2016, 2018, and 2019 (Figure 7).

### 3.3 Nonlinear Effects of Hydrology

**FIGURE 8:**
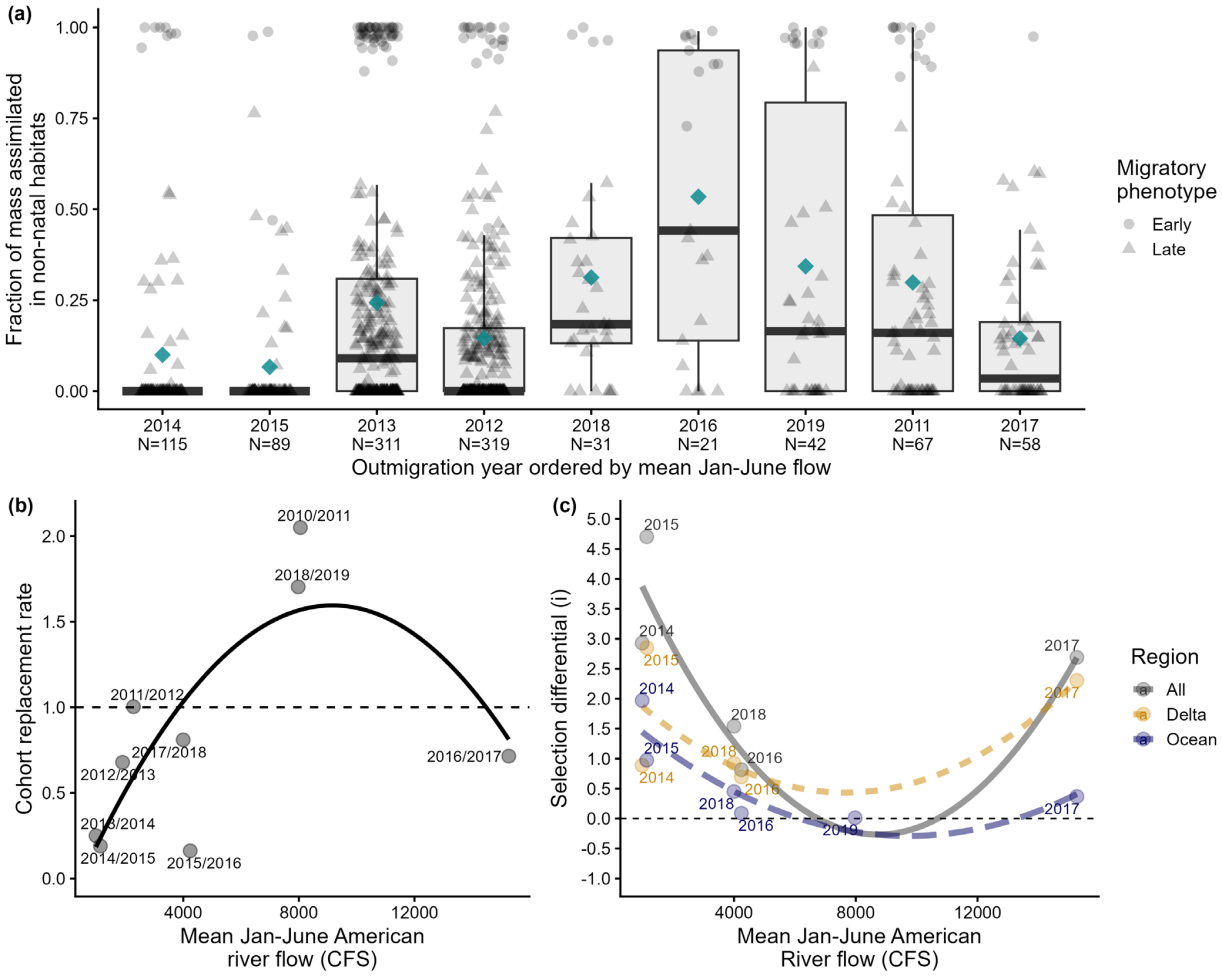
Quadratic relationships with mean Jan‐June river flows: (a) Among‐cohort variation in the fraction of freshwater mass (from emergence to exit from freshwater) assimilated in non‐natal habitats, with years ordered by mean flow. Median and mean fractions indicated by bold lines and diamonds, and early and late migratory phenotypes by circles and triangles, respectively; (b) estimated cohort replacement rates of natural origin fish (no. of returns divided by no. of spawners, after removing hatchery fish), labeled by the parents' spawning year and offspring outmigration year (i.e., the value of 0.7 for 2016/2017 suggests that for every adult that spawned in fall 2016–whose offspring experienced the high flows of winter 2017—approximately 0.7 adults returned in 2018, 2019 or 2020). Rates above or below 1 suggest positive or negative population growth, respectively; (c) standardised selection differentials (*i*) in the Delta (orange, short dashed line), Ocean (blue, long dashed line) and All (grey, solid line), on the basis of the natal exit size of juvenile outmigrants entering vs. leaving the Delta, leaving the Delta vs. returning to spawn, and entering the Delta vs. returning to spawn, respectively. The higher the value, the stronger the size‐selective mortality, with positive values indicating relatively lower survival of smaller outmigrants.

In the adult returns (where we could be confident their otoliths included their full freshwater growth history), we observed large individual and interannual variation in the relative mass assimilated in the natal stream vs. the freshwater Delta (Figure 8). Although most individuals assimilated more mass within the natal river (annual mean ranging from 47%–93%), in every cohort, some non‐natal rearing was observed and there were always examples of early migrants that assimilated most (> 90%) of their ‘freshwater mass’ in non‐natal habitats, ranging from 2% (2017) to 29% (2016) of individuals per year (Table S3). Overall, the mass assimilated in non‐natal habitats exhibited a quadratic relationship with freshwater flows, being lowest in drought years 2014 and 2015, and extreme wet year 2017 (means = 10%, 7% and 14% respectively), and highest in intermediate flow years 2016 and 2018 and wet year 2019 (means = 53%, 31% and 34%, respectively) (Figure 8).

**FIGURE 9:**
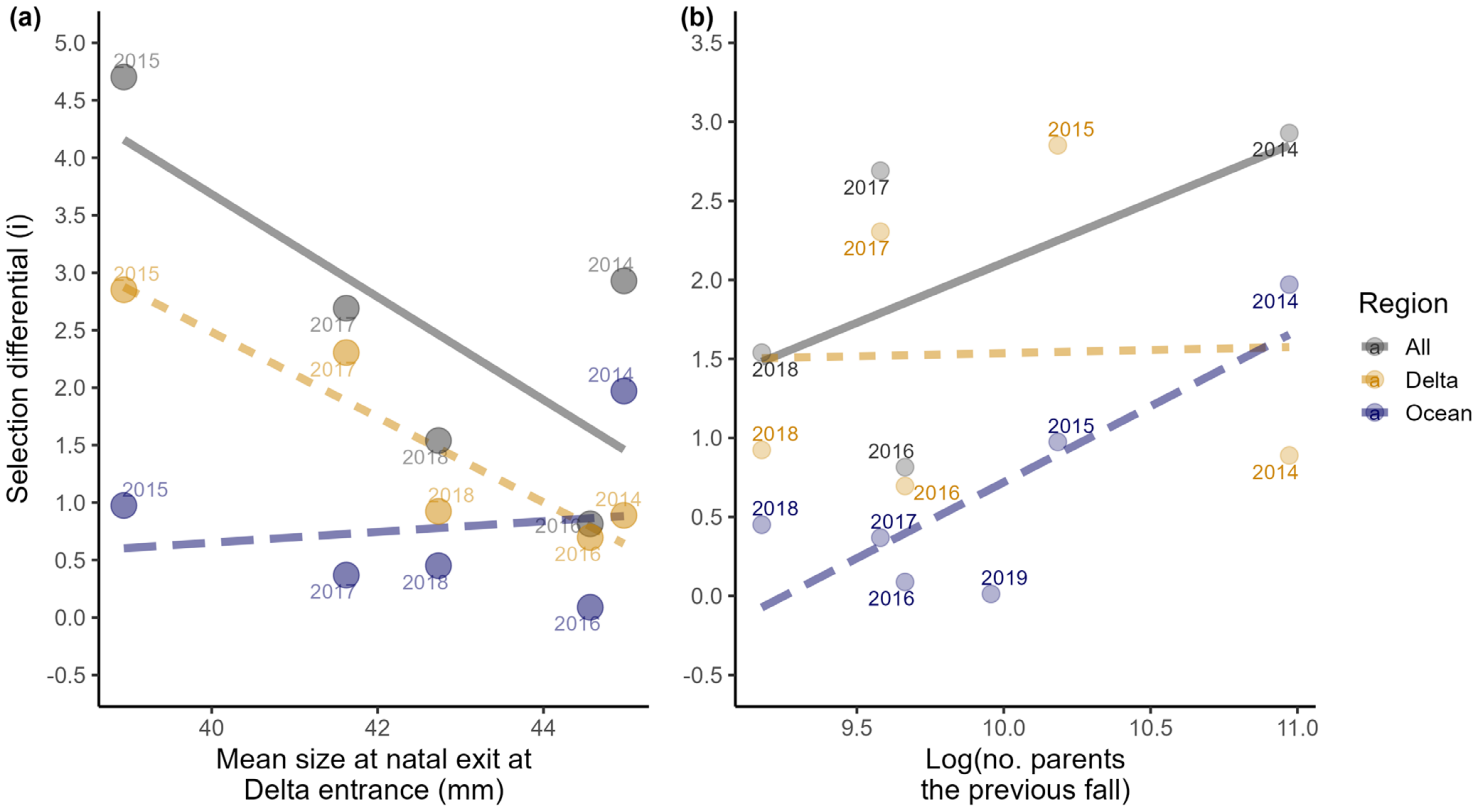
Relationships between selection differentials and (a) mean size at natal exit at the earliest sampling point and (b) total juvenile production, inferred by the logged number of parent spawners the previous fall, for the Delta (orange, short dashed line), Ocean (blue, long dashed line) and ‘All’ (grey solid line), on the basis of the natal exit size of juvenile outmigrants entering vs. leaving the Delta, leaving the Delta vs. returning to spawn, and entering the Delta vs. returning to spawn, respectively.

Selection on size was generally about two times higher in the Delta than the Ocean (Figure 8), with standardised selection differentials (*i*) averaging 1.53 vs. 0.64, respectively. The lowest observed selection differentials were 0.013 and 0.089, indicating basically no change in the natal exit size of fish sampled leaving the Delta vs. returning to spawn in 2019 and 2016, respectively. Conversely, the highest values were observed during the drought, reflecting a ~2 times increase in mean natal exit size between the juveniles entering vs. leaving the Delta (2015) and entering the Delta vs. returning to spawn (2014 and 2015; Figure 7). Although non‐significant (all *p* > 0.05), selection differentials appeared to exhibit quadratic relationships with mean river flows for all phases of the life cycle (Figure 8). Selection on size was strongest in drought years and in the extreme flood year 2017, and weakest in intermediate to wet years. Selection on size in the Delta was also stronger when fish entered the Delta smaller (*F*_1,3_ = 17.9, *p* = 0.024; Figure 9a), and stronger in the Ocean when densities were likely higher, inferred by total escapement the previous fall (*F*_1,4_ = 7.69, *p* = 0.050; Figure 9b).

Focusing only on adult returns (i.e., spawner to spawner abundances using independent datasets)—cohort replacement rates of natural origin fish also showed a quadratic‐like relationship with freshwater flows during the juvenile outmigration period (*F*_2,6_ = 4.68, *p* = 0.0597), being lowest for outmigration years 2015 and 2016 (0.19 and 0.16, respectively), and more than two times higher for wet outmigration year 2011 (2.04) *cf*. extreme wet year 2017 (0.71) (Figure 8).

Finally, although not the primary focus of this paper, annual hydrological conditions were also associated with escapement size and hatchery contribution rates, with the total number of spawners in 2010–2021 (years with paired hatchery stray rates) ranging from 9663 in 2017 (the main return year from drought year 2015) to 58,228 in 2013 (the main return year from wet year 2011) (Table S4). The fraction of spawners that were hatchery origin—on the basis of CFM‐ and otolith‐based assignments (Table S4)—ranged from 32% to 95%, increasing by about 25% in years before vs. after the drought (mean = 58% ± 14% SD in escapement years 2010–15 vs. 83% ± 13% in 2016–21, respectively) (Figure S10).

## 4 Discussion

As land and streamscapes have been homogenised to more efficiently serve human activities, their resilience to stressors such as hydroclimate extremes is reduced, with adverse impacts on ecosystems and humans alike (Moore and Schindler 2022). Climate extremes are projected to become more frequent and severe in the future (Swain et al. 2018), with CCV droughts predicted to increase in severity and duration (Cloern et al. 2011), impacting fish conservation, agriculture, and domestic water supply. Water security and agriculture have driven most infrastructural changes in the Delta, which has been transformed from a rich wetland into cultivated lands intersected by simplified and engineered channels that transport water across California quickly and efficiently to support agriculture and cities (Robinson et al. 2014). Resulting habitat loss, along with introduced piscivores, contaminants, and warming, has resulted in extremely low juvenile salmon survival in the Delta (Buchanan et al. 2018), particularly during dry years (Michel 2018; Michel et al. 2015; Sturrock et al. 2015, 2020). Here, drought years 2014 and 2015 were associated with almost no successful Delta rearing, the strongest size‐selective mortality, both in the Delta and Ocean, and the lowest cohort replacement rates. Wetter years are typically associated with higher Delta survival and growth. However, 2017—the wettest year in the time series—did not follow this pattern. This suggests that extreme flows can also be associated with elevated mortality. As discussed further below, we suggest that interannual variation in cohort strength is strongly influenced by variation in the survival and rearing opportunities available to early migrants, an issue that will be most acute for rivers lower in the system—like the AME—that drain directly into the Delta and thus have fewer options downstream, and in systems—like the CCV—where wetlands and floodplains have been decimated (Munsch et al. 2022). Habitat restoration along the full migratory corridor will be critical to buffer future impacts of ‘whiplash weather’, and to support the full suite of salmon life histories through this period of rapid hydroclimatic change (Swain et al. 2025).

### 4.1 The Effects of Climate Extremes on Juvenile Salmon

Many relationships in nature are non‐linear, particularly with regards to salmon responses to flow and restoration actions (Munsch et al., 2020). Here, with the quadratic relationships between river flow and non‐natal growth, size selection, and return rates observed here somewhat analogous to the bell‐shaped productivity trends predicted by the ‘Intermittent Upwelling Hypothesis’ (Menge and Menge 2013). Importantly, the selective forces at play—and thus the associated management implications—are very different for the two flow extremes. The negative effects of drought on juvenile salmonid survival are well documented. Warming is a major driver of large‐scale declines in Pacific salmonids (Crozier et al. 2021), with Chinook salmon being a cold‐adapted species distributed from California to the Arctic, whose growth performance decreases rapidly at temperatures greater than 20°C (Perry et al. 2015). Also, under hot, dry conditions, the increased metabolic rates of piscivores such as black bass (*Micropterus* spp.) can result in nonlinear increases in predation rates, with juvenile salmon survival rates through the Delta predicted to be near‐zero at water temperatures > 20°C (McInturf et al. 2022; Nobriga et al. 2021). Low flows and high temperatures are also associated with increased redd superimposition and pre‐spawning mortality, reduced early migration and thus increased crowding and competition in natal reaches, as well as reduced prey production and connectivity (Sturrock et al. 2020, 2022; Tillotson and Quinn 2017).

**FIGURE 10:**
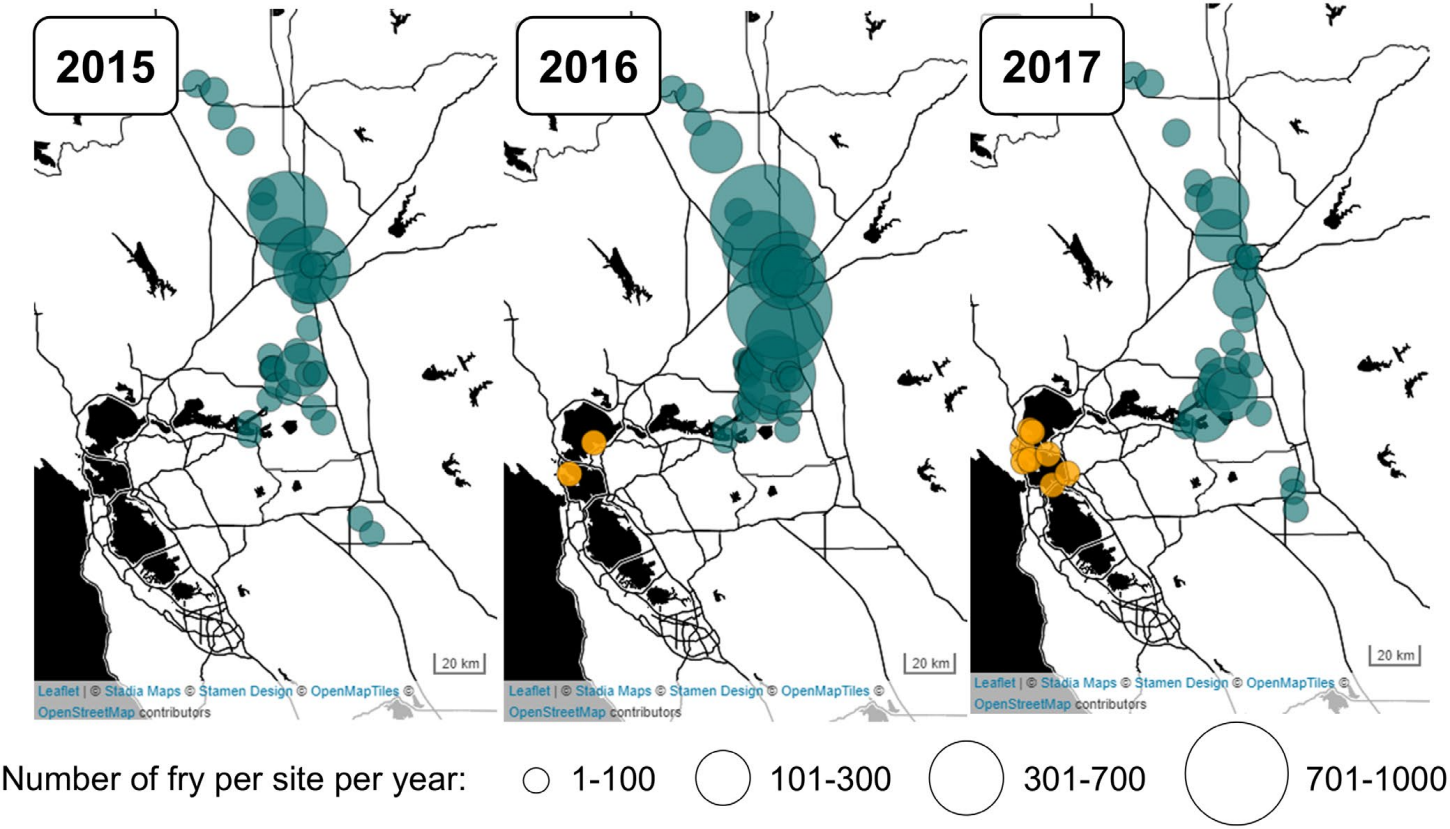
Showing the increasing spread of fry downstream at increasing flows, from drought year 2015, intermediate flow year 2016, to flood year 2017. Early migrants caught in the Bay are highlighted in orange. Specifically, circles represent the raw counts of unmarked Chinook salmon < 55 mm FL (i.e., not expanded for flow, size, or gear interactions, nor unmeasured fish and using the size threshold from Willmes et al. 2024) caught from annually monitored beach seine sites, separated by water year (between October 1 of the year prior to Oct 1 of the year labelled).

Generally, higher river flows are associated with increased overbank flooding and floodplain reconnection, and improved growing conditions for juvenile salmon as a result of increased invertebrate production and subsidies, and thermal and habitat refugia (Cordoleani et al. 2022; Jeffres et al. 2008, 2020; Sommer et al. 2001). Indeed, only positive relationships between river flow and cohort replacement rate were observed in Sturrock et al. (2020), however, peak daily flows in that time series (34,600 cubic feet per second (CFS) in April 2006) were less than half of those seen in this study (82,476 CFS in February 2017). Here, atmospheric rivers in January and February 2017 resulted in two massive flood events that approached a 50‐year flood flow in the natal river. This likely caused redd scouring (Fairman 2007) and downstream transport of yolk sac fry that are known to be weak swimmers (Thomas et al. 1969). This was evidenced by high numbers of newly emerged fry (mean size = 40.2 mm) sampled in the Bay in January to March 2017, as far out as the Golden Gate Bridge (Figure 10). Historically, high flows would have been spread among the complex, interconnected channels and floodplain wetlands of the Delta, attenuating peak velocities and extending inundation duration (Robinson et al. 2014). However, these habitats have almost disappeared today, with over 90% of historical wetlands lost and most riverine and delta habitats converted to highly simplified canals built to efficiently convey water and provide flood protection (Dahl 1990). When flows increase in these simplified channels, water velocities also increase, advecting fry far downstream.

The size at which the surviving adults had entered brackish waters was unimodal and centered around 80 mm FL, a pattern that has proven to be remarkably consistent for subyearlings in the CCV across years, populations, and runs (Cordoleani et al. 2021; Phillis et al. 2018; Sturrock et al. 2020; Willmes et al. 2024). This suggests strong physiological control on salt readiness and a minimum size for successful smoltification for Chinook salmon in this system (Beamish and Mahnken 2001). In 2017, during the critical February to March rearing period, fry in the outer Bay—that normally depend on continued freshwater growth—were exposed to a rapid increase in salinity, from 6.5 PSU on February 20th to > 20 PSU on March 11th at Alcatraz Island (USGS gauge no. 374938122251801; Figure S11). We hypothesise that fry displaced into the outer Bay during the high winter flows of 2017 experienced low survival as a result of osmotic shock and reduced growth (Morgan and Iwama 1991), low habitat quality and quantity (Robinson et al. 2014), and influxes of marine and estuarine predators with the shifting halocline (Bastos et al. 2014). Indeed, we found almost no evidence of successful estuarine rearing in the otolith samples, with only a single juvenile and no returning adults exhibiting otolith strontium concentrations indicative of extended periods of growth in brackish water (Figure 3B). Reaching the minimum size threshold for smoltification would have also been challenging for early migrants that managed to hold territory within the Delta in 2017, with this year characterised by particularly high losses of fry at the Central Valley and State Water Projects (Figure 1, Table 3). Indeed, the estimated mortality of early migrants (unmarked fall run salmon ≤ 47 mm from any population for both water projects combined) was 19,141 individuals for 2017 (*cf*. 0–55 individuals per year in intermediate flow years 2012–2018; Table 3) that were all caught in January to March (Figure S13). Although some of this mortality will have resulted from direct entrainment, the estimates also reflect high predation rates in the waters around the facilities, which are extremely difficult to estimate accurately (Jahn and Kier 2020), particularly for early migrants that are too small to tag.

**TABLE 3:** Estimated annual mortality of unmarked (adipose fin intact) length‐at‐date fall run juvenile salmon at the water export facilities (CVP and SWP; Figure 1) for all years in the time series, separated by size.

Year	Estimated mortality of early migrants (≤ 47 mm FL)	Estimated mortality of late migrants (> 47 mm FL)
2011	637	33,642
2012	0	2322
2013	48	8602
2014	0	401
2015	9	17
2016	50	155
2017	19,141	20,541
2018	55	14,587
2019	3724	8785

*Note:* Years represent ‘water years’ (Oct 1st of year prior to Oct 31st of the expressed year). Note that hatcheries were not releasing fry during these years, so the early migrants should be entirely natural origin, but the late migrants will be a mixture of hatchery and natural origin fish, as ~ 75% of hatchery fish were released unmarked. Estimates from SacPAS, accessed 21st December 2025 (https://www.cbr.washington.edu/sacramento/data/query_loss_detail.html).

Given that early migrants can represent the majority of Chinook salmon outmigrants from natal rivers each year (Sturrock et al. 2020), even small decreases in their survival can have large impacts on cohort strength, as suggested by the lower cohort return rate in 2017 compared to other wet years. Conversely, for fry that held territories in natal streams during the 2017 winter storms, the high flows and overbank flooding were likely beneficial, potentially extending the thermal rearing period (Merz and Vanicek 1996; Munsch et al. 2022), providing high densities of invertebrate prey (Jeffres et al. 2020), increasing emigration size due to longer residence times and/or faster growth (Sellheim et al. 2025), and increasing diversity in ocean arrival timings.

### 4.2 Chasing Ghosts Using Otoliths and Strategic Sampling

Size‐dependent mortality can bias estimates of somatic growth rates and compromise the accuracy of fisheries stock assessments (Goodyear 2019). By randomly sampling a single population and cohort over time, otoliths provide a unique means to quantify the direction and magnitude of size‐ and growth‐selective mortality across regions and time periods. Similar methods have previously been applied to estimate survival rates of alternative migratory phenotypes in the Stanislaus River (Sturrock et al. 2020) (Figure 1). In that study, the upstream sample was obtained from a calibrated RST situated at the confluence of the natal river, enabling the estimation of abundance and phenotype‐specific survival rates (Zeug et al. 2014). Similar to this study, strong size‐selective mortality was observed for the Stanislaus population, acting primarily against small, early migrants; however, it was not possible to pinpoint the mortality hotspot and whether it was focused in the Delta or Ocean. Here, by adding in a strategic sampling point at the freshwater‐marine transition point, it was possible to identify the Delta as the main ‘pinch point’. That said, selection on natal exit size was also important in the ocean in most years, but particularly during the drought years 2014 and 2015. Elevated ocean mortality rates of early migrants are likely explained primarily by their smaller size at ocean entry (Sogard 1997), but potentially also by other carryover effects such as higher contaminant loads and/or stress resulting from their extended residence times in the Delta (Lehman et al. 2017). Higher ocean mortality rates would also be expected for the earlier part of the time series, with changing wind patterns in winter 2014 resulting in an extreme marine heatwave and food web shift off the northern US and Canada nicknamed as “The Blob” (Brodeur et al. 2019; NOAA 2019). Possible environmental factors contributing to the size‐selective mortality observed in the ocean in 2017 and 2018 are less clear, but the period of early ocean residence is well documented as being a sensitive period for salmonids, with many studies showing selection on size during this critical period (Claiborne et al. 2011; Sogard 1997; Woodson et al. 2013), representing the main argument for hatcheries releasing juveniles at larger sizes (Huber and Carlson 2015).

### 4.3 The Importance of Environmental Diversity for Supporting Diverse Migration Strategies

The benefits of maintaining diverse portfolios of habitats, environmental niches, and migratory behaviors for supporting salmon resilience have been shown both theoretically (Schindler et al. 2015) and empirically (Brennan et al. 2019), with forming a central component of the Portfolio Effect (Schindler et al. 2010; Makhlouf et al. 2025). The results of this study support the need to maintain a diverse portfolio of life history strategies to support resilient populations in the face of growing environmental instability. Although early migrants are often assumed to have negligible survival through the Delta, otoliths have revealed that they consistently contribute to reproduction (Cordoleani et al. 2021, 2024; Phillis et al. 2018; Sturrock et al. 2015, 2020; Willmes et al. 2024). That said, in this study, the outmigration year characterised by the highest fraction of early migrants and Delta rearing (2016) was paired with the lowest cohort return rate (0.16 returns per spawner). Whether the two observations are related is unclear, but it may have been that cohort strength was set within the natal river even prior to fry emergence. Indeed, the parents contributing to this cohort spawned during some of the lowest river flows in the time series (fall 2015; Figure 5), being at the tail end of a five‐year drought, so the reduced return rate for this cohort could have resulted from redd superimposition and low egg survival.

Importantly, the contribution rates of early migrants to the adult spawners vary considerably among years and populations, representing 7%–23% of returning spawners in the Stanislaus River, 33%–89% in the Yuba River, and 10%–52% in the AME (using the same size cutoff for cross‐population comparability, Table 2). The high variation in contribution rates will be partly explained by variations in phenotype expression (i.e., downstream movement timing), which is modulated by natal river size and carrying capacity, winter flow magnitude and timing, and fry densities (Apgar et al. 2021; Sturrock et al. 2020), and partly by survival through downstream freshwater habitats and the ocean. The spatial configuration of the river network and the quality and quantity of freshwater habitats downstream of the natal river will play a critical role in governing downstream survival, particularly for early migrants that need to find refuge and food to grow and undergo the costly process of smoltification. Indeed, the natal river with the highest contribution rates of early migrants to the adult returns (the Yuba River) is located upstream of a rich mosaic of freshwater habitats, including the Feather, Bear, and Sacramento Rivers, multiple smaller creeks, and two large managed floodplains (Willmes et al. 2024). For salmon‐producing rivers with shorter migratory corridors (e.g., the Stanislaus, Mokelumne, and American in the CCV, which all have impassable dams preventing access to headwaters and drain directly to the Delta, Figure 1), habitat restoration and reconnection in the natal stream and along the full migration route will be particularly beneficial. Indeed, there is ample evidence that restoration can increase in‐river survival, growth, and rearing duration of juvenile Chinook salmon (Blankenship et al. 2024; Sellheim et al. 2016) and that marine survival is often positively associated with size and growth (Sogard 1997). Therefore, a primary goal of combined flow and non‐flow actions should be to increase juvenile salmon growth before they leave freshwater to improve their long‐term survival. Although management actions often focus on natal habitats, the data herein also emphasise the importance of restoring and reconnecting habitats within the Delta. Flow actions could also improve salmon growth and survival in less productive parts of the Delta and Bay by boosting food supply, transporting valuable prey items from upstream floodplains that are otherwise inaccessible to many populations (Sturrock et al. 2022). However, further research is required to design reservoir releases and restoration actions at appropriate locations and scales to maximise benefits to target species and life stages across a range of hydrologic conditions, while ensuring that sufficient water is retained in reservoirs to meet ecological objectives during future droughts (Null et al. 2024).

### 4.4 Is the Delta an Ecological Trap?

An ecological trap occurs when animals continue to use habitats that lower their fitness relative to other available habitats following rapid environmental change (Hale and Swearer 2016). Historically, the Delta was a vast, productive wetland of dendritic channels and complex habitats, rich with invertebrates and refugia for salmon fry. Sportfish such as striped and largemouth bass were introduced to California in the late 1800s, and over the last 150 years, Delta wetlands have been drained and replaced with riprap‐reinforced, streamlined channels to support agriculture and optimise water transfers (Robinson et al. 2014; Munsch et al. 2022). Because of physiological limits on salt tolerance, early migrants need to remain in the Delta for several months until they reach about 75 mm and become salt‐ready. During this time, they face high predator densities, limited habitat and food, elevated contaminants, and large water exports that entrain juvenile salmon (Lehman et al. 2017; Jahn and Kier 2020) (Table 3). Our results suggest the Delta somewhat functions as an ecological trap for early migrants, which experience high mortality both in the Delta and ocean. These findings support targeted restoration of Delta habitats for early migrants, paired with improvements in natal rivers to increase carrying capacity and enable increased growth prior to entering the Delta gauntlet.

Although early migrants were a primary focus of this paper, the Delta may also be an ecological trap for late migrants, particularly during drought. Given hatchery smolts are often trucked to the Bay (circumventing the Delta altogether), this can augment survival imbalances between hatchery and natural origin juveniles, as suggested by the *c*.25% increase in the fraction of hatchery origin fish spawning in the AME following the 2014–15 drought (Figure S10; discussed further in Text S2). Juvenile salmon survival rates in freshwater appear to be governed by a delicate tradeoff between size, time, and temperature, with selection on size generally favoring larger juveniles until water temperatures—and the metabolic rates of warm‐adapted piscivores—start to increase in late spring, when survival rates of even the largest fish drop rapidly (Nobriga et al. 2021; Scheuerell et al. 2009; Sturrock et al. 2020). Drought years in the CCV are also characterised by truncated outmigration periods (Munsch et al. 2022), slower growth (Coleman et al. 2022), and apparently lower survival of subyearlings (*cf*. yearlings that leave in fall) (Cordoleani et al. 2021).

Study limitations and management recommendations are discussed further in Text S3, but—in brief—in order to fully understand the extent to which the Delta represents an ecological trap and to design appropriate management actions such as habitat enhancement or reservoir releases, managers need to know (a) how river flow and habitat carrying capacity interact to influence phenotype expression (i.e., the prevalence of early migration vs. fry remaining upstream), and (b) the survival rates of fry in the natal river vs. the Delta at different densities and environmental conditions. These metrics require annual production estimates (i.e., the number of fry successfully emerging from the gravel), as well as efficiency‐corrected abundance estimates for the two trawl sites (the RST is situated too far upstream to use for this purpose). Unfortunately, such abundance data were unavailable, so we estimated *relative* size‐selective mortality through the Delta and Ocean, and were unable to compare fry mortality within the natal river vs. downstream. However, the cohort replacement rates provided an independent measure of total survival that did incorporate in‐river mortality. Another limitation is that interpreting phenotype fractions without paired abundance data for each sampling point carries inherent uncertainties. For example, the surprising loss of early migrants in 2017 could have theoretically reflected minimal change in their survival rate and disproportionately high survival of late migrants. However, the weight of evidence—historically high flow rates during the fry emergence period, lack of observed Delta rearing, high numbers of fry caught at the water projects and in the outer Bay just before a sharp rise in salinity, and an overall reduced cohort replacement rate—suggest a nontrivial loss of early migrants.

Finally, tracking a single population within a mixed‐stock sample poses inherent challenges, particularly regarding sample size requirements. In the CCV, the high numbers of unmarked hatchery fish (usually representing 75% of all releases) further increased these requirements, sometimes resulting in low sample sizes for certain sites and years (Table 1). To circumvent some of these issues, we also estimated total selection differentials (natal exit to return), given that these two sampling points were generally characterised by larger sample sizes. Additionally, we used RST data from the lower AME to estimate juvenile size distributions entering the Delta in 2014 and 2015, given no (2014) or unbalanced (2015) sampling by the Sherwood Harbor trawl in these years. Sensitivity analyses suggest that the resulting interannual patterns are robust (Figure S8) and the range of *i* values observed here overlapped those reported by Perez and Munch (2010) for other fish species; however, the absolute selection differentials for the Delta and ‘All’ in 2014 and 2015 may be somewhat overestimated given the RST location being 9 miles upstream of the confluence and the isotopic breakpoint used to define Delta entry in the survivors. Finally, separating AME and Nimbus Hatchery fish (late migrants) using otolith ^87^Sr/^86^Sr alone proved difficult, so we incorporated additional natal markers, including the exogenous feeding check score and eye lens δ^34^S, which added considerable time and cost. As eye lens δ^34^S prescreening for escapement years 2013–16 was not possible, potential ‘contamination’ of Nimbus Hatchery fish in our AME‐assigned fish is likely higher in these years; however, the large drop in natural‐origin spawners post‐drought was observed the year before lens prescreening began (2017), and estimated numbers of hatchery fish on the spawning grounds did not differ using otolith‐ vs. CFM‐derived estimates (Figure S10), increasing confidence in the resulting assignments. If all hatchery fish were externally marked, reconstructing the growth, behavior, and survival of natural‐origin individuals would be far easier. Recent studies investigating the survival rates of hatchery‐produced fry increasingly rely on parentage‐based tagging (Pope et al. 2024).

## 5 Conclusion

Given the complex habitat needs of migratory species, management actions need to consider the spatial and temporal configuration of different stressors, how they carry over across habitats and life stages, and how they influence population size and stability. Here, all phenotypes contributed to the spawning population in all years, emphasizing the importance of maintaining a diverse life history portfolio. However, large‐scale loss of freshwater wetlands and degradation of other complex habitats along the migratory corridor have reduced non‐natal rearing opportunities that would have historically supported salmon and provided refugia during extreme flows. Downstream habitat mosaics are particularly important for populations located lower in the system, such as the American River. In a rapidly changing climate, we need to embrace a multi‐tool approach to pinpoint mortality hotspots and to understand how selective pressures vary across land‐, river‐ and seascapes, which can be highly dynamic for transboundary migratory species such as salmon. By monitoring changes in trait distributions and survival we can design targeted and climate‐ready management and conservation actions that promote viable and resilient fish populations.

## Author Contributions

**Anna M. Sturrock:** conceptualization, data curation, formal analysis, funding acquisition, investigation, methodology, project administration, visualization, writing – original draft, writing – review and editing. **Carson Jeffres:** writing – review and editing. **George Whitman:** investigation, writing – review and editing. **Jamie Sweeney:** data curation, investigation, methodology, resources, writing – review and editing. **Joseph Merz:** conceptualization, funding acquisition, investigation, methodology, project administration, resources, writing – review and editing. **Kohma Arai:** formal analysis, investigation, methodology, visualization, writing – review and editing. **Kirsten Sellheim:** conceptualization, funding acquisition, investigation, methodology, project administration, resources, writing – original draft, writing – review and editing. **Malte Willmes:** formal analysis, investigation, methodology, writing – review and editing. **Miranda Bell‐Tilcock:** investigation, methodology, writing – review and editing. **Rachel C. Johnson:** conceptualization, funding acquisition, investigation, methodology, project administration, resources, writing – review and editing.

## Funding

This work was supported by the California Department of Fish and Wildlife, P1596028. U.S. Fish and Wildlife Service, F21AP01966. UK Research and Innovation, MR/V023578/1.

## Conflicts of Interest

The authors declare no conflicts of interest.

## Supporting information


**Text S1:** Further method details.
**Text S2:** Hatchery impacts.
**Text S3:** Recommendations and study limitations.
**Figure S1:** Fork length distribution of adults sampled in this project showing the thresholds used to retrospectively assign fish that did not have scale reads as 2‐year‐olds (< 57 cm) or 4‐year‐old (> 100 cm) returns on the basis of known‐aged hatchery returns on other rivers.
**Figure S2:** Eye lens δ^34^S distributions by return year, including all samples screened, with vertical lines showing the assumed thresholds for wild (≤ 10.5‰) vs. hatchery origin fish (> 10.5‰), although otoliths from fish with lens δ^34^S values > 16‰ were still analysed to check for potential estuarine rearing.
**Figure S3:** Assignment accuracy of known‐origin American River natural origin smolts (AME) and Nimbus Hatchery smolts (NIH) after assigning them using the model in Arai et al. (2026), here represented as ‘baseline’, then *post hoc* adjusting NIH‐assigned fish with mean exogenous scores > 1.5 to AME if the posterior probability to NIH was < 0.6, < 0.7, < 0.8 or < 0.9.
**Figure S4:** All otolith strontium isotope profiles of unmarked adult fish sampled on the American River spawning grounds in 2013–2021 assigned as natural origin American River (AME), strays from Nimbus Hatchery (NIH), which is situated on the American River, or strays from other rivers or hatcheries.
**Figure S5:** Size threshold used to define early vs late migration (321 μm otolith radius, equivalent to 47 mm FL) as defined by the breakpoint between modes using (a) natal exit sizes reconstructed in the otoliths from all fish included in this study. (b) The 47 mm threshold (red dashed line) seemed to reflect the valley between size modes of juveniles sampled by rotary screw trap (RST) in the lower American River in 2013‐19 (PSMFC, 2014) (abundances estimated using https://github.com/tmcd82070/CAMP_RST). (c) The FL data from the RST shows consistent operation in the two years used (2014–2015), when Nimbus also did not release hatchery fish upstream of the trap.
**Figure S6:** (a) Broken stick regression line (red) used to convert otolith radius (OR) into fork length (FL) in juvenile Chinook salmon in this study (updated from Sturrock et al. 2020 and Willmes et al. 2024) based on 813 juveniles of known size and OR (Equation 1 in main text). (b) Shows the relationship used to predict wet (thawed) mass from otolith radius (Equation 2 in main text).
**Figure S7:** Differences between catch distributions of juvenile Chinook salmon (length‐at‐date fall run, unmarked; green) and the fraction sampled for otoliths (purple) by (a) 1‐month, (b) 3‐month, and (c) 4‐month time bins. Given the unbalanced otolith sampling at Sherwood Harbor in 2015 and absent sampling from this site in 2014, we used RST data to estimate the size distribution of fish entering the Delta in these 2 years. Sometimes there were insufficient samples within a single time bin, in which case it was combined with the subsequent time bin.
**Figure S8:** Sensitivity analysis to explore the implications of different juvenile data on the (A) proportion of early vs late migrants, (B) natal exit size distributions at sequential sampling points, and (C) relationship between selection differentials and mean river flow. Top left: raw unmodified trawl data. Top right: RST size distributions used to replace the missing or patchy years for the Sherwood Harbor trawl (2014–2015; Fig. S7). Subsequent figures show the effect of expanding (resampling) the otolith samples from trawl sites to mimic observed catch distributions for Sherwood Harbor and Chipps (left) or Sherwood Harbor only (right) within 1‐, 3‐, or 4‐monthly time bins.
**Figure S9:** (a) shows the number of hatchery fish spawning in the American River between 2014 and 2019 estimated using otolith and eye lens chemistry (this study) vs coded wire tags (CWT; CFM reports). Line of best fit with 95% CI shown in black and shaded grey, 1:1 line shown as dashed red line. (b) shows the estimated cohort replacement rates obtained using hatchery fractions from (a) and assuming 100% of adults returned age‐3.
**Figure S10:** (a) Total numbers of hatchery (red) vs. wild (teal) spawners per year, estimated by otolith assignment for 2014–2021 and CFM for 2010–2013. (b) The fraction of hatchery origin spawners each year, fitted with a loess smoother ± 95% CI. Years representing returns before vs. after the 2014–2015 drought are separated by the red dashed line, with return year 2016 represented primarily by 3‐year‐olds that outmigrated in 2014 and 2‐year‐olds that outmigrated in 2015.
**Figure S11:** Mean daily salinity near Golden Gate Bridge in the San Francisco Bay between Jan 1st and June 30th 2011–2019. Data downloaded from USGS (Alcatraz Island gauge, site number 374938122251801) on 18th February 2025.
**Figure S12:** Estimated loss of Chinook salmon at the SWP and CVP water export facilities in 2017, from https://www.cbr.washington.edu/sacramento/data/delta_salvage.html (accessed December 21st 2025), with the fall run early migrants circled in red.
**Table S1:** Sample sizes of juvenile salmon per site, region (relative to the Delta), collection method, and year, separated between no. assigned to the American River (AME) vs. other sources (non‐AME).
**Table S2:** Instrument operating conditions of the Nu Plasma HR (Nu032) and New Wave Research UP213 Nd:YAG 213 nm laser.
**Table S3:** Fraction of freshwater mass assimilated in non‐natal habitats (mean, median and standard deviation) and fraction of individuals that achieved > 90% of their growth in natal or non‐natal habitats.
**Table S4:** Estimated numbers of hatchery and natural origin fish contributing to the total escapement estimated using otoliths and eye lens chemistry (this study) vs. coded wire tag (CWT) returns by the Constant Fractional Marking Program (CFM).

## Data Availability

The code, data and figures that support the findings of this study are openly available in Figshare at https://doi.org/10.6084/m9.figshare.31871866.
