# Supplementary material for: A Modern Ghost Story: Increased Selective Mortality of Salmon Under Climate Extremes
Source: Glob Chang Biol. 2026 Apr 28;32:e70854. doi: 10.1111/gcb.70854 (PMC13122436; doi:10.1111/gcb.70854)
Supplement: Supplementary file 1 — Text S1: Further method details. Text S2: Hatchery impacts. Text S3: Recommendations and study limitations. Figure S1: Fork length distribution of adults sampled in this project showing the thresholds used to retrospectively assign fish that did not have scale reads as 2‐year‐olds (< 57 cm) or 4‐year‐old (> 100 cm) returns on the basis of known‐aged hatchery returns on other rivers. Figure S2: Eye lens δ34S distributions by return year, including all samples screened, with vertical lines showing the assumed thresholds for wild (≤ 10.5‰) vs. hatchery origin fish (> 10.5‰), although otoliths from fish with lens δ34S values > 16‰ were still analysed to check for potential estuarine rearing. Figure S3: Assignment accuracy of known‐origin American River natural origin smolts (AME) and Nimbus Hatchery smolts (NIH) after assigning them using the model in Arai et al. (2026), here represented as ‘baseline’, then post hoc adjusting NIH‐assigned fish with mean exogenous scores > 1.5 to AME if the posterior probability to NIH was < 0.6, < 0.7, < 0.8 or < 0.9. Figure S4: All otolith strontium isotope profiles of unmarked adult fish sampled on the American River spawning grounds in 2013–2021 assigned as natural origin American River (AME), strays from Nimbus Hatchery (NIH), which is situated on the American River, or strays from other rivers or hatcheries. Figure S5: Size threshold used to define early vs late migration (321 μm otolith radius, equivalent to 47 mm FL) as defined by the breakpoint between modes using (a) natal exit sizes reconstructed in the otoliths from all fish included in this study. (b) The 47 mm threshold (red dashed line) seemed to reflect the valley between size modes of juveniles sampled by rotary screw trap (RST) in the lower American River in 2013‐19 (PSMFC, 2014) (abundances estimated using https://github.com/tmcd82070/CAMP_RST). (c) The FL data from the RST shows consistent operation in the two years used (2014–2015), when Nimbus also did not release h [file GCB-32-e70854-s001.pdf]

Supplemental Material

A modern ghost story: Increased selective mortality of salmon under climate extremes

Anna M. Sturrock, Kirsten Sellheim, Joseph Merz, Jamie Sweeney, Miranda Bell-Tilcock, George Whitman, Kohma Arai, Malte Willmes, Carson Jeffres, Rachel C. Johnson

Text S1. Further method detail

Eye lens pre-screening: For knowns and unknown origin fish, one eye lens per fish was delaminated and all laminae between diameters of 800-1200 μm were combined, dried and encapsulated in pressed tin capsules and analyzed for bulk $\delta^{34}S$ at the UC Davis Stable Isotope Facility using the methods outlined in Bell-Tilcock et al. (2021). Separation in natal lens $\delta^{34}S$ was observed using known AME wild (mean = 7.64 ± 2.30 SD, range = 5.25-10.5‰, n=5) and Nimbus Hatchery (mean = 13.28 ± 1.33 SD, range = 11.04-14.43‰, n=5) juveniles, resulting from the marine-derived fish meal in the hatchery feed (*cf.* freshwater invertebrates being the main dietary source for natural origin fish), suggesting a hatchery vs. wild threshold value of 10.5‰.

Sensitivity analyses for trawl expansions: For our sensitivity analysis to explore the impacts of alternative expansion scenarios to match the otolith sample to the observed catch at that trawl site, we randomly resampled otoliths with replacement within each time bin, using the catch fraction to weight the otolith sample. To check that the expansions were the same independent of the time bin used, we repeated this exercise using a variety of sampling scenarios, resampling both Chipps and Sherwood Harbor samples vs. only Sherwood Harbor samples (given that this was the key site where early migrants were clearly undersampled in 2015), and testing the effect of using (c) 'monthly' time bins (in reality, some months needed to be combined in order to have at least 5 otoliths per time bin, but all were 1-3 months), (d) 3-monthly and (e) 4-monthly time bins (Fig. S7 and Fig. S8).

Text S2. Hatchery impacts

Here, we saw clear drought effects on the contribution rate of hatchery-origin fish to natural spawning grounds, with a *c.*25% increase in the fraction of hatchery origin fish in returns after the 2014-15 drought. This is likely due to a combination of reduced survival of natural origin juveniles during droughts and drought-related changes in hatchery practices, in particular increases in the numbers of fish trucked directly to the estuary (Sturrock et al., 2019). The impacts of such high numbers of hatchery fish on natural spawning grounds could be multifaceted. Blankenship et al. (2024) found an inverse relationship between probability of spawning success and adult abundance in the AME, suggesting density dependent impacts which may be driven by spawning/redd disruption or limited habitat or prey resources for emerging juveniles. Since up to 90% of in-river spawning fish were of hatchery origin, selection

pressure from hatchery production is the dominant force shaping the Chinook Salmon population. Hatchery-produced juvenile Chinook Salmon have been documented to demonstrate less variable migration strategies, whether due to hatchery practices (e.g., release timing and location) or behavioral differences between wild and hatchery fish (Knudsen et al., 2006; Takata et al., 2017; Willmes et al., 2018). Addressing hatchery impacts will require changes in hatchery management that explicitly support the maintenance of diverse life history strategies while simultaneously addressing limitations in river rearing habitat to improve success of natural origin juveniles.

Text S3. Recommendations and study limitations

Since at least 72% of historic Chinook Salmon habitat in the CCV has been lost due to impoundments (Yoshiyama et al., 2001) and remaining habitat is severely degraded due to historic and current mining and urban and agricultural expansion, it is critically important that remnant habitat be expanded and maintained to support natural spawning and rearing. Climate extremes are projected to become more frequent and severe in the future (Swain et al., 2018), yet restoration efforts are often designed around a relatively narrow range of ‘average’ conditions and fail to consider habitat function and life stage specific survival patterns (Battin et al., 2007; Beechie et al., 2013). Expanding off-channel rearing habitat that functions under a range of flow conditions is essential for retaining the natal rearing strategy and supporting increased survival rates across the entire migratory corridor. However, without selection differentials within the natal river (i.e. from spawning grounds to natal exit) to compare the Delta values to, or better yet, absolute survival rates in-river vs. Delta, it would be challenging to use the data in this study alone to guide dam operators and restoration managers in where best to target their efforts. Ultimately, we would recommend restoration both upstream and downstream combined with a series of flow release experiments.

If survival of fry is lower in the Delta than the natal river, a first step could be to take actions that reduce expression of the early migration strategy (i.e., delay emigration). Early migration is likely triggered by a combination of winter flow cues (Zeug et al., 2014) and crowding (Sturrock et al., 2020). Therefore, one might argue to reduce pulse flows from the reservoir in January to March and to increase carrying capacity of upstream habitats through habitat enhancement. However, in years with high fry production, this could have unintended consequences by concentrating fry within a small area, leading to increased in-river density dependent mortality, which can be exacerbated under lower flow conditions (Sturrock et al., 2020). If the carrying capacity of the natal river influences the prevalence of early migration via density dependent migration (Greene & Beechie, 2004), one might argue to focus habitat restoration in the natal stream to reduce expression. If we assume that early migration is inevitable and the consequence of genetic predisposition and/or winter storm events (whether triggered or transported), one might argue for increased Delta restoration and potentially flow pulses in March and April to transport prey downstream to support them. In the absence of other data, the fact that early migrants consistently contributed to the adult spawning population and - in other rivers - in-river carry capacity can be limiting (Sturrock et al., 2020), it might be prudent to mimic the natural

hydrograph rather than to suppress winter flows that might trigger early migration. Particularly for years with high numbers of spawners (which is known by end of the calendar year) winter pulse flows could help to reduce density dependent mortality by spreading fry through the system (Yarnell et al., 2015). However, it is less clear whether this would be an advisable strategy during drought years, when - at least during this study - selection against early migrants was high, both in the Delta and Ocean.

The Delta Juvenile Fish Monitoring Program has been performing long term trawl surveys at Sherwood Harbor and Chipps Island since the 1970s (Interagency Ecological Program 2023). Although these methods provide a measure of relative density and timing of outmigration for the CCV salmon stock complex across years, most restoration efforts occur at the river level, and it is only recently that genomics is enabling differentiation of different runs and populations, although fall run remain genetically homogenous (Thompson et al., 2024). Otolith methods provide a valuable opportunity to assign individuals to river of origin in order to estimate population specific juvenile production. However, we were hampered by low sample sizes, lack of abundance estimates and being swamped by unmarked hatchery fish. We recommend increased, stratified otolith sampling at both trawl sites, developing more rapid hatchery vs. wild diagnostics (assuming 100% marking is not a possibility), and developing trawl efficiency models to expand catches into estimated abundances of AME origin fish at both sampling points to support Delta and Ocean survival rates. To estimate in-river survival rates one would need reliable fry production estimates, possibly with a rotary screw trap situated immediately below the spawning grounds (similar to the upstream trap on the Stanislaus River; Zeug et al. 2014). The existing rotary screw trap on the AME is too far downstream to estimate fry production and it would be better if it could placed closer to the confluence to produce reliable estimates of outmigrant abundance. In addition, Nimbus Hatchery often releases unmarked smolts upstream, and backwater effects from high Sacramento River flows frequently reduce trap function. Finally, the trap does not operate during AME flows greater than ~10,000 CFS due to safety concerns, which meant that we could not use the RST to estimate size distributions in years other than 2014 and 2015. However, the low flow conditions in 2014-15 meant that the traps were operated throughout the season, and as no hatchery fish were released upstream, we felt more confident in using the RST size distributions for these two cohorts.

Supplemental references

Bell-Tilcock, M., Jeffres, C. A., Rypel, A. L., Sommer, T. R., Katz, J. V. E., Whitman, G., & Johnson, R. C. (2021). Advancing diet reconstruction in fish eye lenses. *Methods in Ecology and Evolution*, *12*(3), 449–457. https://doi.org/10.1111/2041-210X.13543

Blankenship, S. M., Scherer, A., Dean, C., Sellheim, K., Sweeney, J., & Merz, J. (2024). Applying parentage methods to detect gravel augmentation effects on juvenile Chinook Salmon recruitment rates. *River Research and Applications*, *40*(5), 791–808. https://doi.org/10.1002/rra.4264

Greene, C. M., & Beechie, T. J. (2004). Consequences of potential density-dependent mechanisms on recovery of ocean-type chinook salmon (Oncorhynchus tshawytscha). *Canadian Journal of Fisheries and Aquatic Sciences*, *61*(4), 590–602. https://doi.org/10.1139/f04-024

Knudsen, C. M., Schroder, S. L., Busack, C. A., Johnston, M. V., Pearsons, T. N., Bosch, W. J., & Fast, D. E. (2006). Comparison of Life History Traits between First-Generation Hatchery and Wild Upper Yakima River Spring Chinook Salmon. *Transactions of the American Fisheries Society*, *135*(4), 1130–1144. https://doi.org/10.1577/T05-121.1

Sturrock, A. M., Satterthwaite, W. H., Cervantes-Yoshida, K. M., Huber, E. R., Sturrock, H. J. W., Nusslé, S., & Carlson, S. M. (2019). Eight Decades of Hatchery Salmon Releases in the California Central Valley: Factors Influencing Straying and Resilience. *Fisheries*, *44*(9), 433–444. https://doi.org/10.1002/fsh.10267

Sturrock, A. M., Carlson, S. M., Wikert, J. D., Heyne, T., Nusslé, S., Merz, J. E., Sturrock, H. J. W., & Johnson, R. C. (2020). Unnatural selection of salmon life histories in a modified riverscape. *Global Change Biology*, *26*(3), 1235–1247. https://doi.org/10.1111/gcb.14896

Takata, L., Sommer, T. R., Louise Conrad, J., & Schreier, B. M. (2017). Rearing and migration of juvenile Chinook salmon (Oncorhynchus tshawytscha) in a large river floodplain. *Environmental Biology of Fishes*, *100*(9), 1105–1120. https://doi.org/10.1007/s10641-017-0631-0

Thompson, T. Q., O'Leary, S., O'Rourke, S., Tarsa, C., Baerwald, M. R., Goertler, P., & Meek, M. H. (2024). Genomics and 20 years of sampling reveal phenotypic differences between subpopulations of outmigrating Central Valley Chinook salmon. *Evolutionary Applications*, *17*(6), e13705. https://doi.org/10.1111/eva.13705

Willmes, M., Hobbs, J. A., Sturrock, A. M., Bess, Z., Lewis, L. S., Glessner, J. J. G., Johnson, R. C., Kurth, R., & Kindopp, J. (2018). Fishery collapse, recovery, and the cryptic decline of wild salmon on a major California river. *Canadian Journal of Fisheries and Aquatic Sciences, Doi: Https://Doi.Org/10.1139/Cjfas-2017-0273*. https://doi.org/10.1139/cjfas-2017-0273

Yarnell, S. M., Petts, G. E., Schmidt, J. C., Whipple, A. A., Beller, E. E., Dahm, C. N., Goodwin, P., & Viers, J. H. (2015). Functional Flows in Modified Riverscapes: Hydrographs, Habitats and Opportunities. *BioScience*, *65*(10), 963–972. https://doi.org/10.1093/biosci/biv102

Yoshiyama, R. M., Gerstung, E. R., Fisher, F. W., & Moyle, P. B. (2001). Historical and present distribution of Chinook salmon in the Central Valley drainage of California. In R. L. Brown (Ed.), *Contributions to the Biology of Central Valley Salmonids, Vol. 1. Fish Bulletin No. 179* (pp. 71–176).

Zeug, S. C., Sellheim, K., Watry, C., Wikert, J. D., & Merz, J. (2014). Response of juvenile Chinook salmon to managed flow: Lessons learned from a population at the southern extent of their range in North America. *Fisheries Management and Ecology*, *21*(2), 155–168. https://doi.org/10.1111/fme.12063

Supplemental Figures

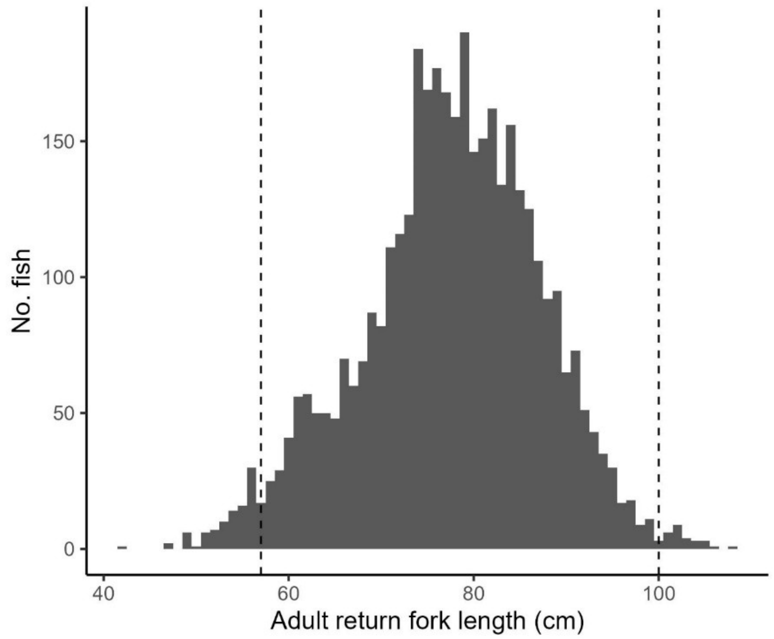

Fig. S1 Fork length distribution of adults sampled in this project showing the thresholds used to retrospectively assign fish that did not have scale reads as two-year olds (<57cm) or four-year old (>100cm) returns based on known-aged hatchery returns on other rivers.

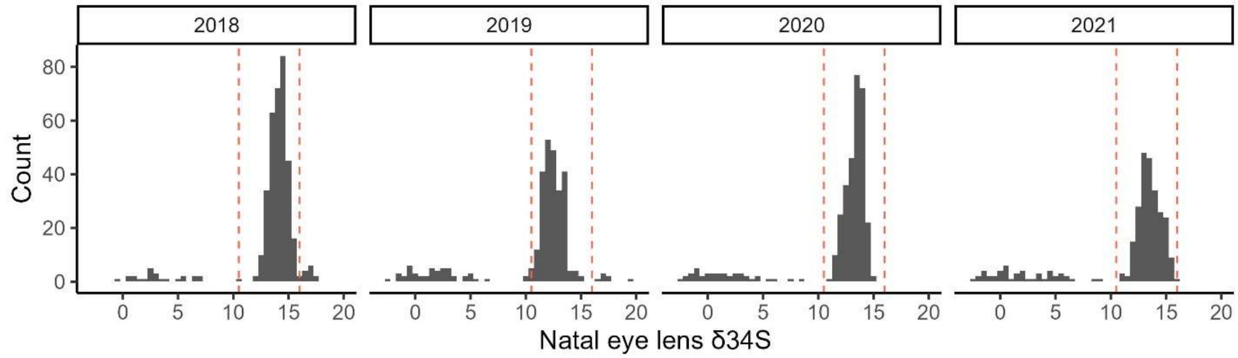

Fig. S2 Eye lens δ^{34}S distributions by return year, including all samples screened, with vertical lines showing the assumed thresholds for wild (≤10.5‰) vs. hatchery origin fish (>10.5‰), although otoliths from fish with lens δ^{34}S values >16‰ were still analysed to check for potential estuarine rearing.

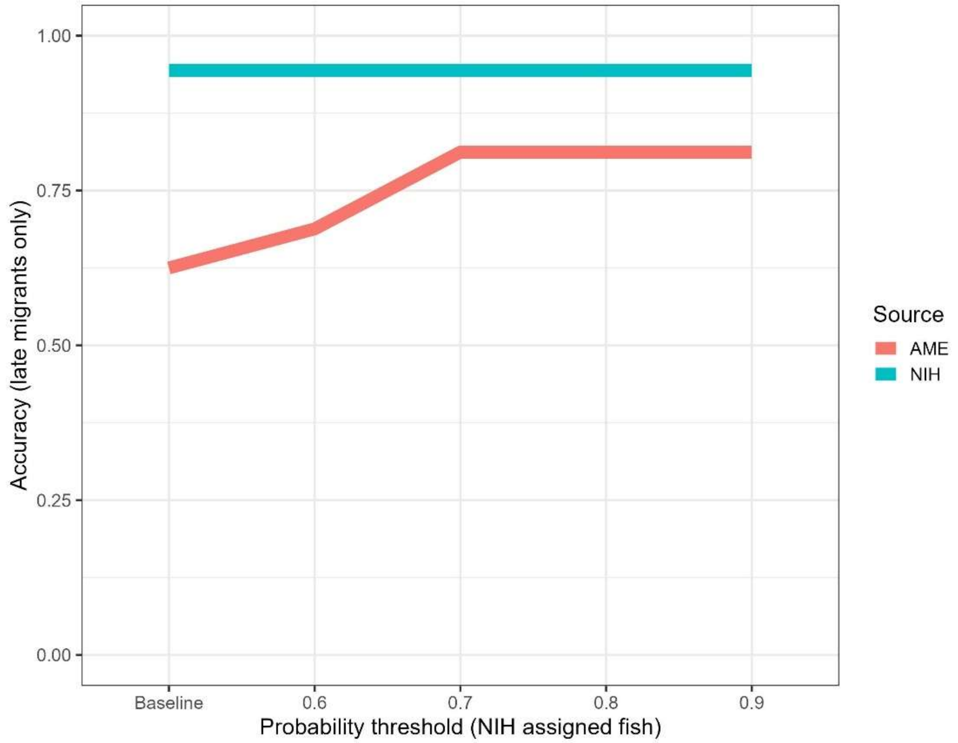

Fig. S3 Assignment accuracy of known-origin American River natural origin smolts (AME) and Nimbus Hatchery smolts (NIH) after assigning them using the model in Arai et al. (2026), here represented as "baseline", then *post-hoc* adjusting NIH-assigned fish with mean exogenous scores > 1.5 to AME if the posterior probability to NIH was <0.6, <0.7, <0.8 or <0.9.

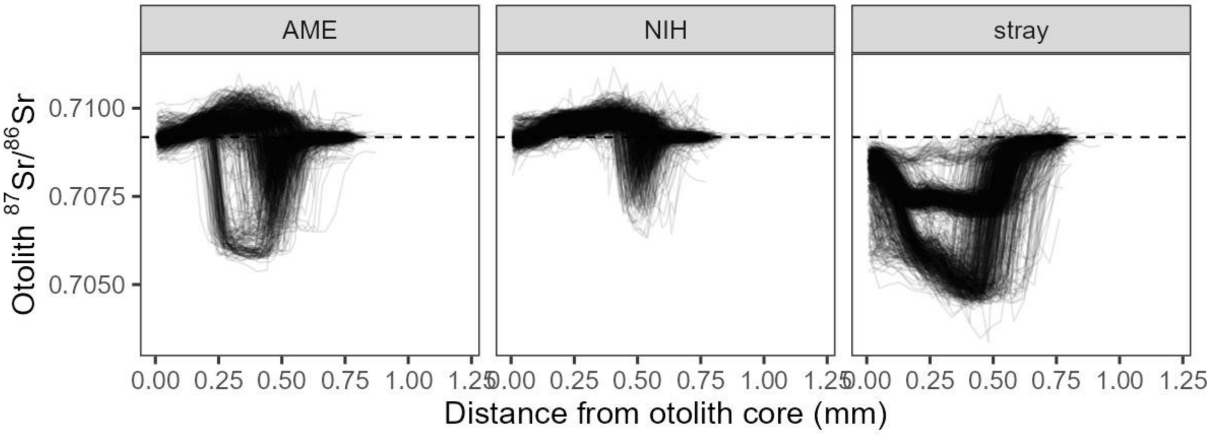

Fig. S4 All otolith strontium isotope profiles of unmarked adult fish sampled on the American River spawning grounds in 2013-2021 assigned as natural origin American River (AME), strays from Nimbus Hatchery (NIH), which is situated on the American River, or strays from other rivers or hatcheries.

(a)

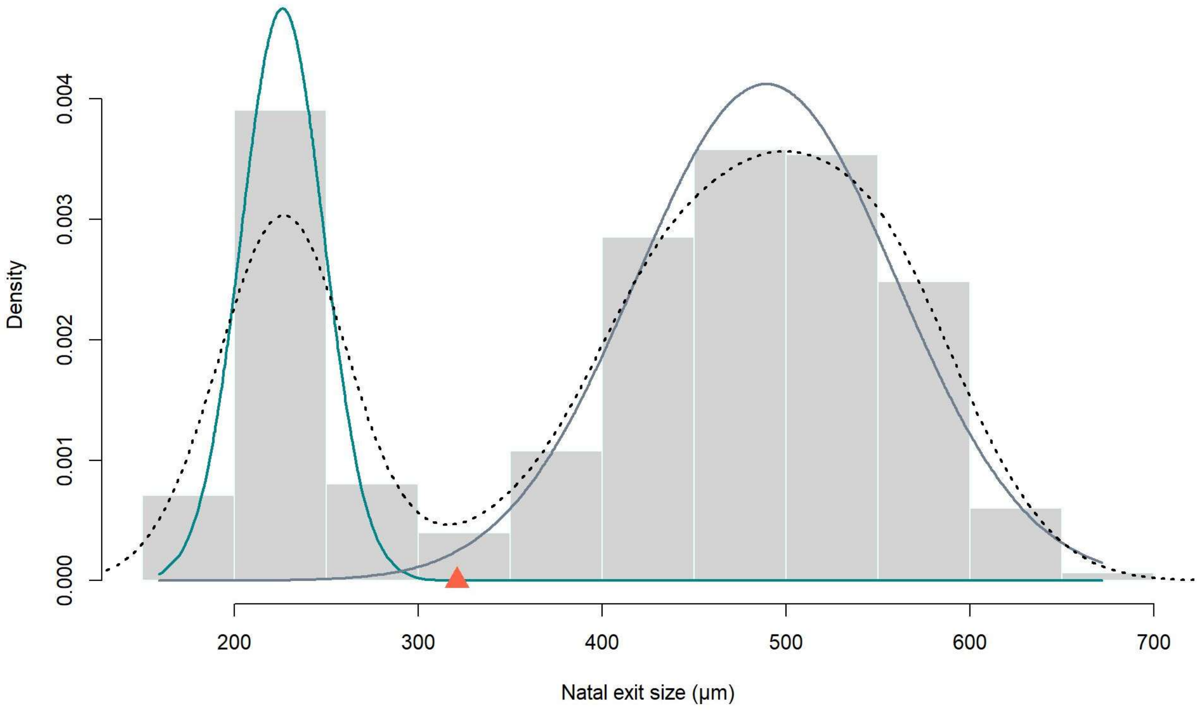

(b)

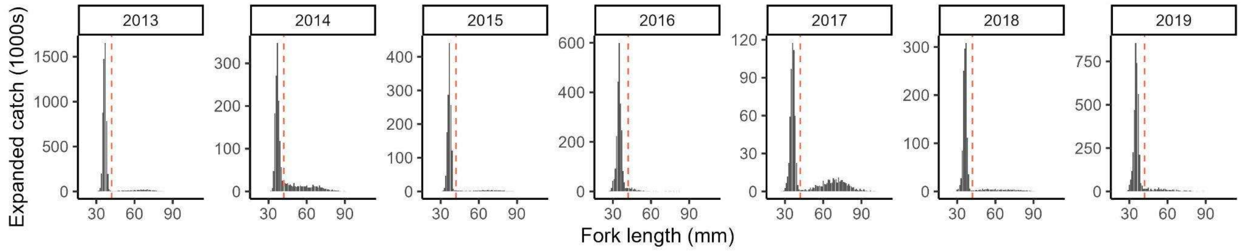

(c)

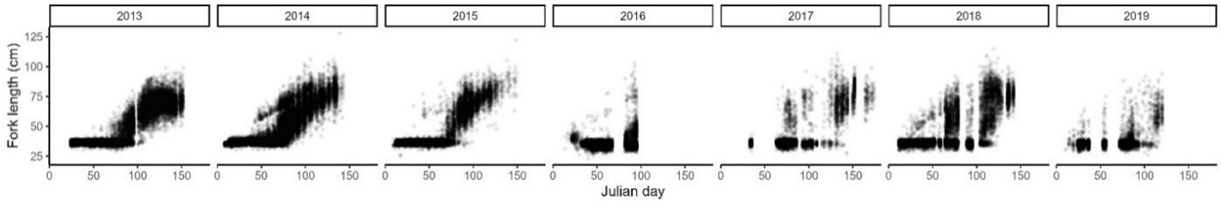

Fig. S5 Size threshold used to define early vs late migration (321 µm otolith radius, equivalent to 47 mm FL) as defined by the breakpoint between modes using (a) natal exit sizes reconstructed in the otoliths from all fish included in this study. (b) The 47 mm threshold (red dashed line) seemed to reflect the valley between size modes of juveniles sampled by rotary screw trap (RST) in the lower American River in 2013-19 (PSMFC, 2014) (abundances estimated using https://github.com/tmcd82070/CAMP_RST). (c) The FL data from the RST shows consistent operation in the two years used (2014-15), when Nimbus also did not release hatchery fish upstream of the trap.

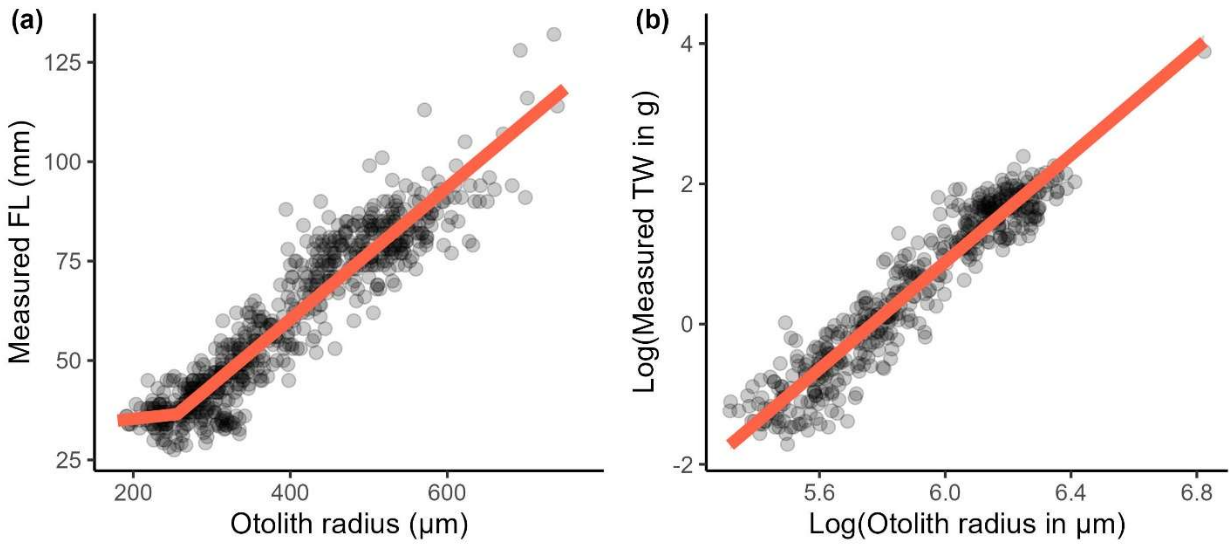

Fig. S6 (a) Broken stick regression line (red) used to convert otolith radius (OR) into fork length (FL) in juvenile Chinook salmon in this study (updated from Sturrock et al. 2020 and Willmes et al. 2024) based on 813 juveniles of known size and OR (Equation 1 in main text). (b) Shows the relationship used to predict wet (thawed) mass from otolith radius (Equation 2 in main text).

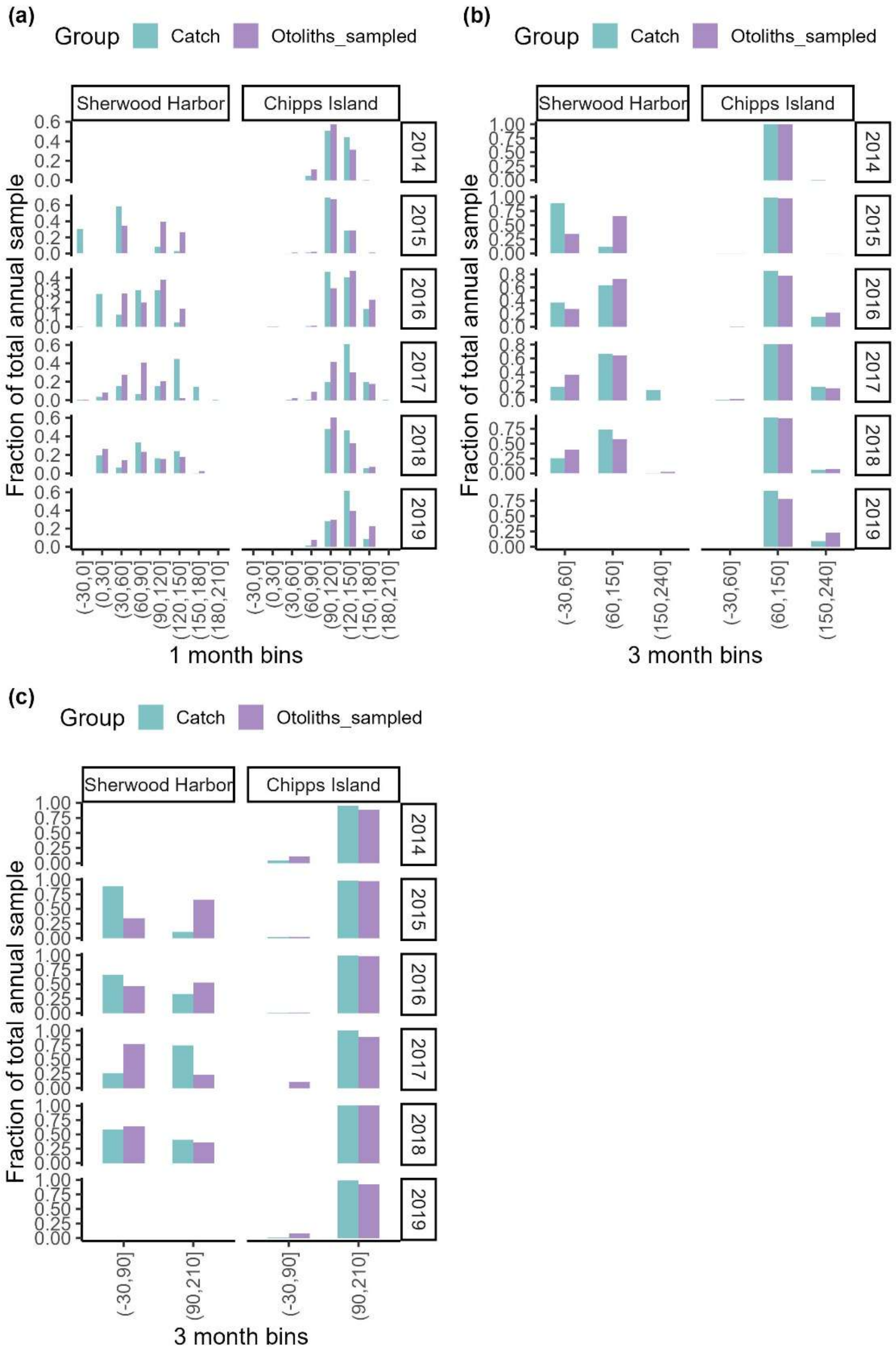

Fig. S7 Differences between catch distributions of juvenile Chinook salmon (length-at-date fall run, unmarked; green) and the fraction sampled for otoliths (purple) by (a) 1-month, (b) 3-month, and (c) 4-month time bins. Given the unbalanced otolith sampling at Sherwood Harbor in 2015 and absent sampling from this site in 2014, we used RST data to estimate the size distribution of fish entering the Delta in these two years. Sometimes there were insufficient samples within a single time bin, in which case it was combined with the subsequent time bin.

Raw trawl data only

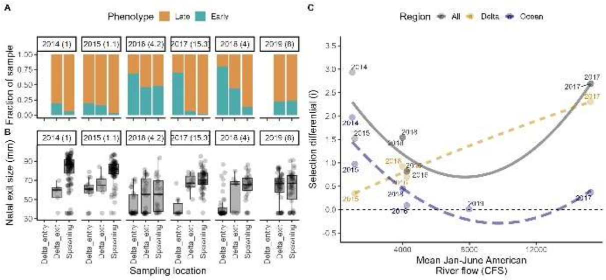

Raw trawl data with RST data for 2014-15

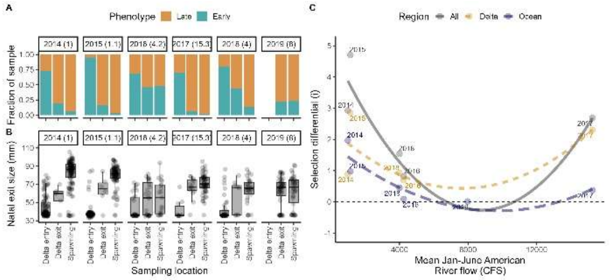

1-monthly expansion Sherwood & Chipps

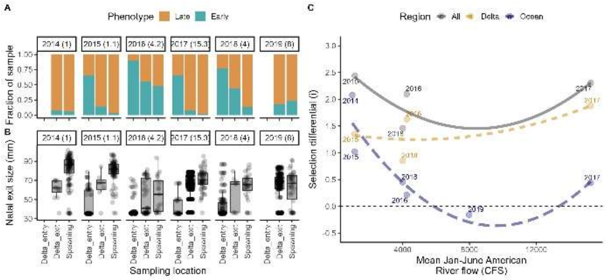

1-monthly expansion Sherwood only

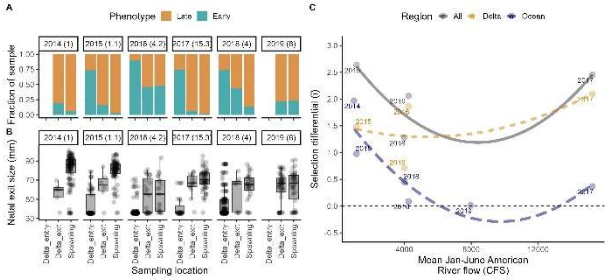

3-monthly expansion Sherwood & Chipps

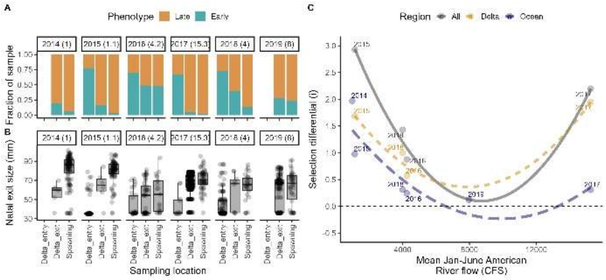

3-monthly expansion Sherwood only

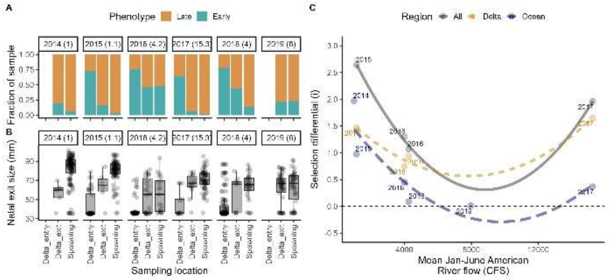

4-monthly expansion Sherwood & Chipps

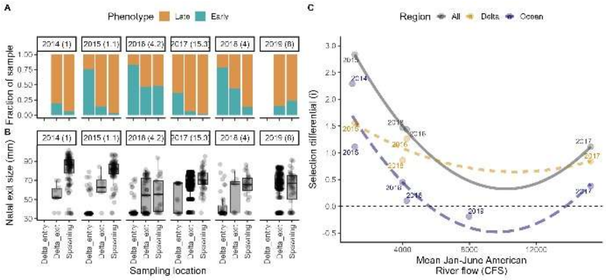

4-monthly expansion Sherwood only

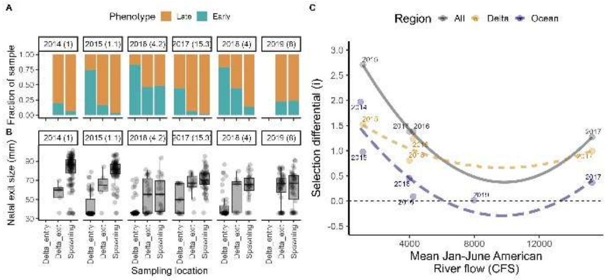

Fig. S8 Sensitivity analysis to explore the implications of different juvenile data on the (A) proportion of early vs late migrants, (B) natal exit size distributions at sequential sampling points, and (C) relationship between selection differentials and mean river flow. Top left: raw unmodified trawl data. Top right: RST size distributions used to replace the missing or patchy years for the Sherwood Harbor trawl (2014-15; Fig. S7). Subsequent figures show the effect of expanding (resampling) the otolith samples from trawl sites to mimic observed catch distributions for Sherwood Harbor and Chipps (left) or Sherwood Harbor only (right) within 1-, 3- or 4-monthly time bins.

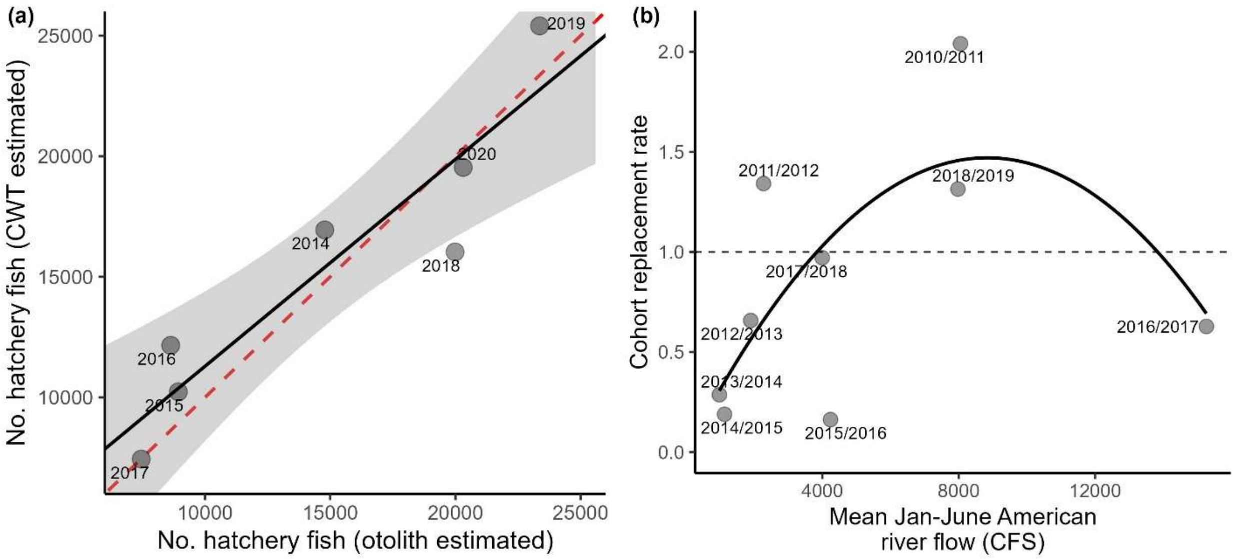

Fig. S9 (a) shows the number of hatchery fish spawning in the American River between 2014 and 2019 estimated using otolith and eye lens chemistry (this study) vs coded wire tags (CWT; CFM reports). Line of best fit with 95% CI shown in black and shaded grey, 1:1 line shown as dashed red line. (b) shows the estimated cohort replacement rates obtained using using hatchery fractions from (a) and assuming 100% of adults returned age-3.

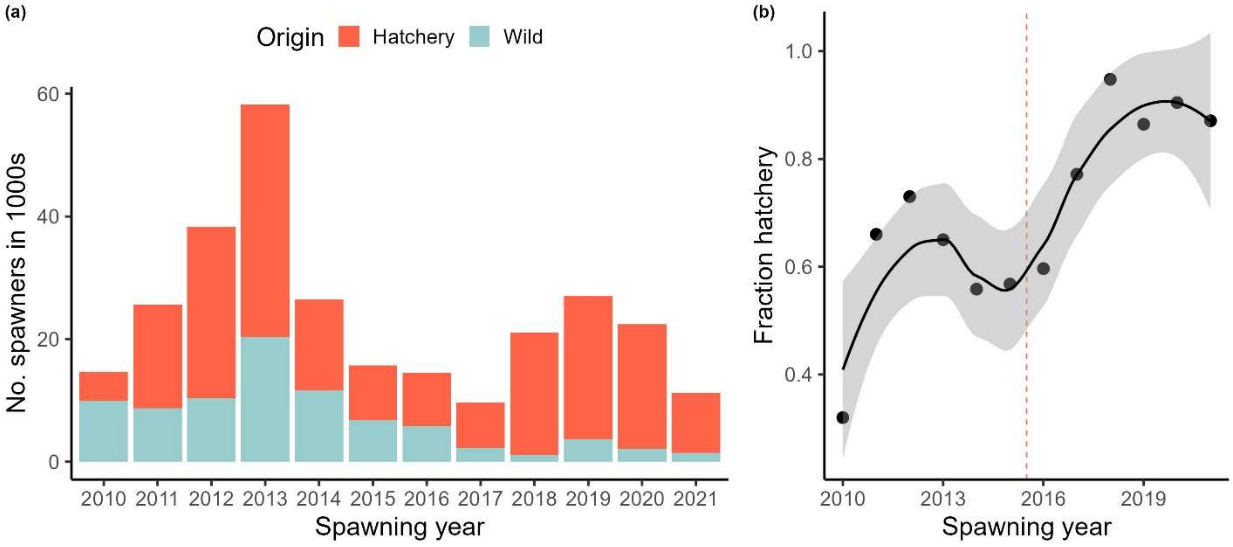

Fig. S10 (a) Total numbers of hatchery (red) vs. wild (teal) spawners per year, estimated by otolith assignment for 2014-2021 and CFM for 2010-2013. (b) The fraction of hatchery origin spawners each year, fitted with a loess smoother ± 95% CI. Years representing returns before vs. after the 2014-2015 drought are separated by the red dashed line, with return year 2016 represented primarily by 3 year olds that outmigrated in 2014 and 2 year olds that outmigrated in 2015.

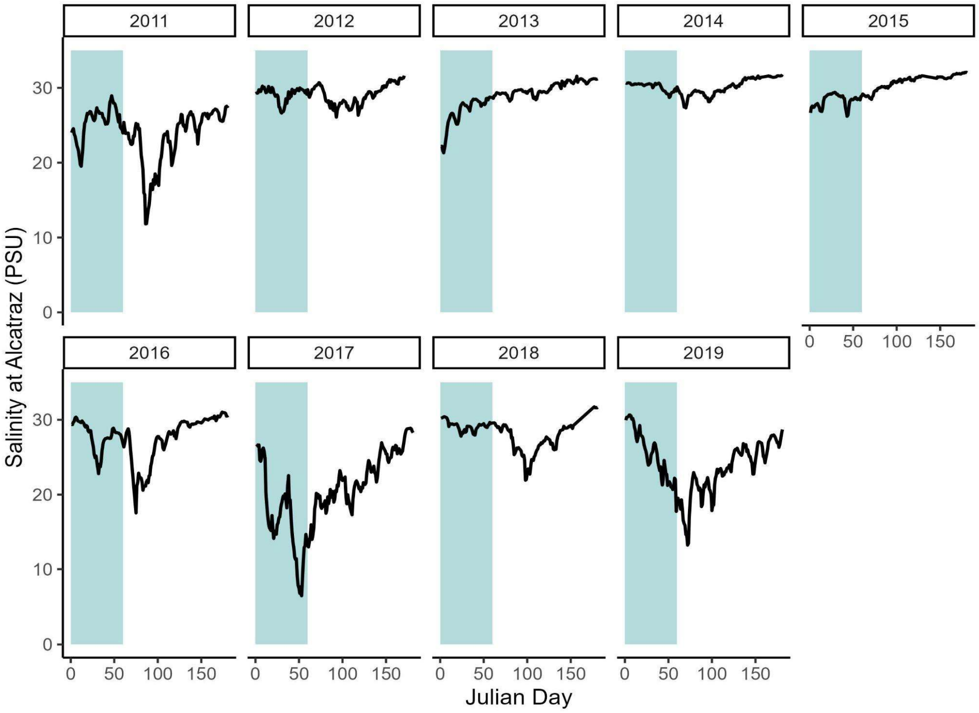

Fig. S11 Mean daily salinity near Golden Gate Bridge in the San Francisco Bay between Jan 1st and June 30th 2011-2019. Data downloaded from USGS (Alcatraz Island gauge, site number 374938122251801) on 18th February 2025.

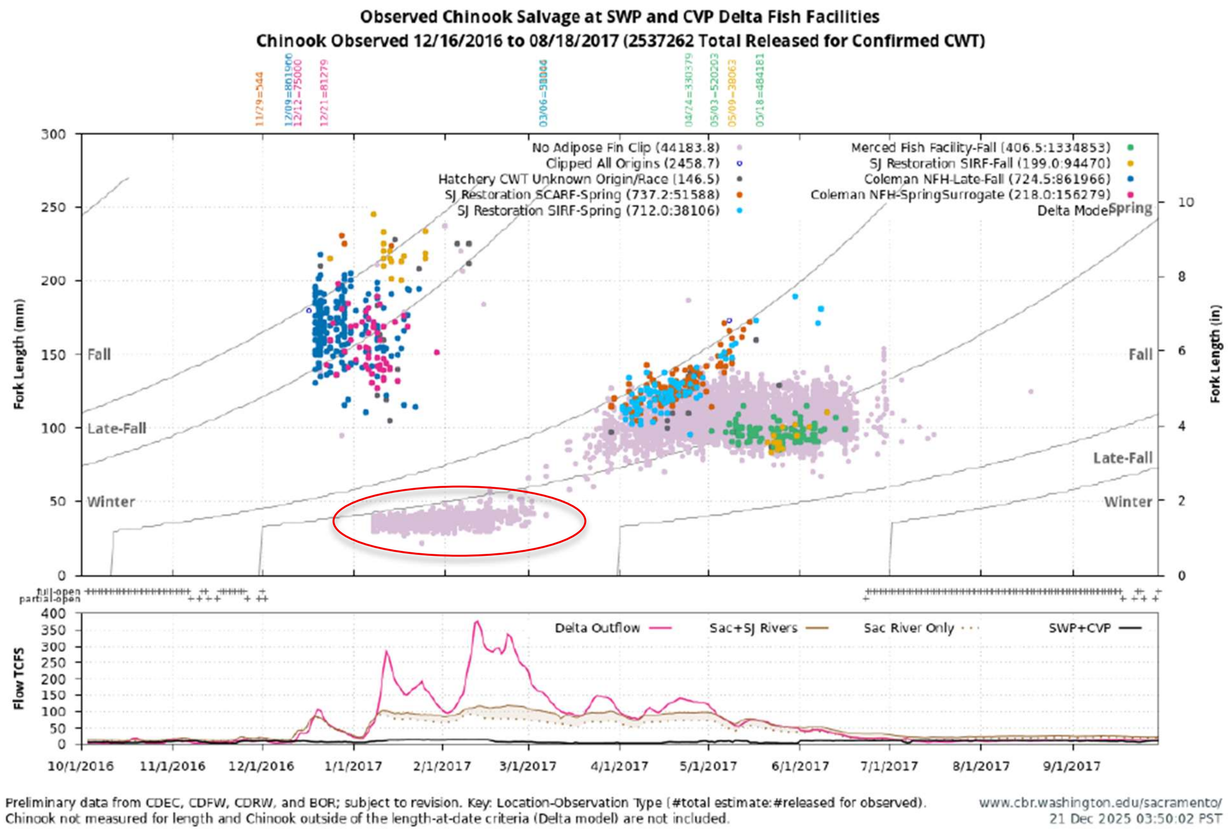

Fig. S12 Estimated loss of Chinook salmon at the SWP and CVP water export facilities in 2017, from https://www.cbr.washington.edu/sacramento/data/delta_salvage.html (accessed December 21st 2025), with the fall run early migrants circled in red.

Table S1. Sample sizes of juvenile salmon per site, region (relative to the Delta), collection method, and year, separated between no. assigned to the American River (AME) vs. other sources (non-AME).

Site Name	Region	Year	Collection method	Latitude	Longitude	N AME	N non-AME
Sherwood Harbor	Upstream	2015	KDTR	38.53279	-121.523	15	23
Sherwood Harbor	Upstream	2016	KDTR	38.53279	-121.523	28	53
Sherwood Harbor	Upstream	2017	KDTR	38.53279	-121.523	10	122
Sherwood Harbor	Upstream	2018	KDTR	38.53279	-121.523	40	37
Chipps Island	Downstream	2014	MWTR	38.04365	-121.911	5	254
Chipps Island	Downstream	2015	MWTR	38.04365	-121.911	6	215
Chipps Island	Downstream	2016	MWTR	38.04365	-121.911	26	304
Chipps Island	Downstream	2017	MWTR	38.04365	-121.911	15	123
Chipps Island	Downstream	2018	MWTR	38.04365	-121.911	9	330
Chipps Island	Downstream	2019	MWTR	38.04365	-121.911	50	212
Paradise Beach	Bay	2016	Seine	37.89413	-122.458	1	0
Berkeley Frontage	Bay	2017	Seine	37.85194	-122.3	1	1
China Camp	Bay	2017	Seine	38.00031	-122.461	0	1
Keller Beach	Bay	2017	Seine	37.92055	-122.387	2	3
McNears Beach	Bay	2017	Seine	37.99242	-122.452	2	2
Paradise Beach	Bay	2017	Seine	37.89413	-122.458	1	0
San Quentin	Bay	2017	Seine	37.94149	-122.482	0	2
Tiburon	Bay	2017	Seine	37.89533	-122.49	0	6
Treasure Island	Bay	2017	Seine	37.81359	-122.37	1	2
Keller Beach	Bay	2018	Seine	37.92055	-122.387	0	4
Paradise Beach	Bay	2018	Seine	37.89413	-122.458	0	1
Antioch Dunes	Within	2014	Seine	38.01481	-121.783	1	13
B&W Marina	Within	2014	Seine	38.12746	-121.58	0	18
Brannan Island	Within	2014	Seine	38.11666	-121.684	0	8
Eddos	Within	2014	Seine	38.05079	-121.699	1	12
Isleton	Within	2014	Seine	38.16273	-121.612	0	3
Koket	Within	2014	Seine	38.24039	-121.555	1	4
Medford Island	Within	2014	Seine	38.05161	-121.51	0	2
Rio Vista	Within	2014	Seine	38.18652	-121.663	1	12
Sandy Beach	Within	2014	Seine	38.13889	-121.695	0	17
Sherman Island	Within	2014	Seine	38.05608	-121.786	1	4
Steamboat Slough	Within	2014	Seine	38.30472	-121.575	0	9
Terminous	Within	2014	Seine	38.10994	-121.5	0	1
Antioch Dunes	Within	2015	Seine	38.01481	-121.783	0	3
Eddos	Within	2015	Seine	38.05079	-121.699	0	1
King Island	Within	2015	Seine	38.05877	-121.458	0	2
Koket	Within	2015	Seine	38.24039	-121.555	3	3
Rio Vista	Within	2015	Seine	38.18652	-121.663	0	3
Sandy Beach	Within	2015	Seine	38.13889	-121.695	0	3
Sherman Island	Within	2015	Seine	38.05608	-121.786	0	1
Steamboat Slough	Within	2015	Seine	38.30472	-121.575	0	1
Terminous	Within	2015	Seine	38.10994	-121.5	0	3

Wimpys	Within	2015	Seine	38.22691	-121.491	1	3
Antioch Dunes	Within	2016	Seine	38.01481	-121.783	1	1
B&W Marina	Within	2016	Seine	38.12746	-121.58	4	31
Brannan Island	Within	2016	Seine	38.11666	-121.684	0	2
Eddos	Within	2016	Seine	38.05079	-121.699	13	5
Isleton	Within	2016	Seine	38.16273	-121.612	0	6
Koket	Within	2016	Seine	38.24039	-121.555	18	9
Medford Island	Within	2016	Seine	38.05161	-121.51	1	10
Rio Vista	Within	2016	Seine	38.18652	-121.663	4	3
Sandy Beach	Within	2016	Seine	38.13889	-121.695	13	15
Sherman Island	Within	2016	Seine	38.05608	-121.786	1	3
Steamboat Slough	Within	2016	Seine	38.30472	-121.575	9	2
Terminous	Within	2016	Seine	38.10994	-121.5	3	13
Wimpys	Within	2016	Seine	38.22691	-121.491	1	3
Antioch Dunes	Within	2017	Seine	38.01481	-121.783	1	10
B&W Marina	Within	2017	Seine	38.12746	-121.58	4	40
Brannan Island	Within	2017	Seine	38.11666	-121.684	0	1
Eddos	Within	2017	Seine	38.05079	-121.699	0	19
Isleton	Within	2017	Seine	38.16273	-121.612	5	13
King Island	Within	2017	Seine	38.05877	-121.458	0	6
Koket	Within	2017	Seine	38.24039	-121.555	3	11
Medford Island	Within	2017	Seine	38.05161	-121.51	0	1
Rio Vista	Within	2017	Seine	38.18652	-121.663	10	23
Sandy Beach	Within	2017	Seine	38.13889	-121.695	3	13
Wimpys	Within	2017	Seine	38.22691	-121.491	1	13
Antioch Dunes	Within	2018	Seine	38.01481	-121.783	1	5
B&W Marina	Within	2018	Seine	38.12746	-121.58	0	2
Isleton	Within	2018	Seine	38.16273	-121.612	4	3
Koket	Within	2018	Seine	38.24039	-121.555	7	4
Medford Island	Within	2018	Seine	38.05161	-121.51	1	1
Rio Vista	Within	2018	Seine	38.18652	-121.663	7	2
Sandy Beach	Within	2018	Seine	38.13889	-121.695	1	5
Sherman Island	Within	2018	Seine	38.05608	-121.786	1	0
Steamboat Slough	Within	2018	Seine	38.30472	-121.575	12	4
Wimpys	Within	2018	Seine	38.22691	-121.491	0	9
Clarksburg	Within	2014	Seine	38.38305	-121.521	2	18
Clarksburg	Within	2015	Seine	38.38305	-121.521	1	6
Clarksburg	Within	2016	Seine	38.38305	-121.521	10	23
Garcia Bend	Within	2016	Seine	38.47805	-121.543	26	14
Clarksburg	Within	2017	Seine	38.38305	-121.521	2	2
Garcia Bend	Within	2017	Seine	38.47805	-121.543	2	7
Sherwood Harbor	Within	2017	Seine	38.52739	-121.529	0	4
Clarksburg	Within	2018	Seine	38.38305	-121.521	4	3
Garcia Bend	Within	2018	Seine	38.47805	-121.543	16	11
**TOTAL**						**413**	**2,197**

Table S2. Instrument operating conditions of the Nu Plasma HR (Nu032) and New Wave Research UP213 Nd:YAG 213 nm laser.

Instrument parameters	
Nu Plasma HR (Nu032) MC-ICP-MS	
Forward power	1300 W
Extraction voltage	6000 V
Analyzer pressure	< 5e-8 mbar
Cones	Nickel dry plasma high sensitivity sampler cone (HS1-9)+ high sensitivity skimmer cone (HS1-7)
Torch depth	5 mm
Detector array	12 Faraday cups, 10^{11} Ω resistors<br>3 Ion Counters
Detector configuration	H4 (88), H2(87), Ax (86), L2 (85),<br>IC0 (104), L3 (84), L4 (83)
Mass resolution	500 (0.3 mm slit)
Gas flows	
Coolant gas	13 L/min
Argon makeup gas	0.85 L/min
Helium gas to cell	0.6 L/min
Auxiliary gas flow	0.85 L/min
New Wave Research UP213 laser	
Nd:YAG	213 nm
Supercell	Low volume laminar flow cell
Laser fluence	3-5.5 J/cm^2
Repetition rate	10 Hz
Spot size	40 µm
Spot spacing	40 µm
Dwell time	20-25 seconds

Table S3. Fraction of freshwater mass assimilated in non-natal habitats (mean, median and standard deviation) and fraction of individuals that achieved >90% of their growth in natal or non-natal habitats.

Outmigration year	Flow category	Mean fraction of FW growth assimilated in non-natal habitats	Median fraction of FW growth assimilated in non-natal habitats	SD in fraction of FW growth assimilated in non-natal habitats	Percent of samples that achieved >90% of their FW growth in natal river	Percent of fish that achieved >90% of their FW growth in non-natal habitats
2011	Wetter	30%	16%	36%	42%	16%
2012	Drier	15%	0%	26%	63%	6%
2013	Drier	24%	9%	35%	51%	16%
2014	Drought	10%	0%	26%	83%	7%
2015	Drought	7%	0%	19%	85%	2%
2016	Wetter	53%	44%	40%	24%	29%
2017	Wetter	14%	3%	21%	52%	2%
2018	Drier	31%	18%	30%	19%	13%
2019	Wetter	34%	16%	40%	40%	24%
All years						

Table S4. Estimated numbers of hatchery and natural origin fish contributing to the total escapement estimated using otoliths and eye lens chemistry (this study) vs. coded wire tag (CWT) returns by the Constant Fractional Marking Program (CFM).

Escapement year	Total escapement (Grand Tab)	Proportion hatchery spawners (this study)	Proportion hatchery spawners (CFM)	Estimated N Hatchery (our study - after marked fish expansion)	Estimated N Hatchery (CFM)	CFM source
2010	14,689		0.32		4,700	Kormos et al 2012
2011	25,626		0.66		16,913	Palmer-Zwahlen and Kormos 2013
2012	38,328		0.73		27,979	Palmer-Zwahlen and Kormos 2015
2013	58,228		0.65		37,848	Palmer-Zwahlen et al 2018
2014	26,475	0.70	0.64	18,466	16,944	Palmer-Zwahlen et al 2019a
2015	15,739	0.63	0.65	9,964	10,230	Palmer-Zwahlen et al 2019b
2016	14,473	0.78	0.84	11,243	12,157	Palmer-Zwahlen and Kormos 2020
2017	9,663	0.82	0.77	7,934	7,441	Letvin et al 2020
2018	21,092	0.95	0.76	19,987	16,030	Letvin et al 2021a
2019	27,030	0.89	0.94	23,740	25,408	Letvin et al 2021b
2020	22,456	0.90	0.87	20,258	19,537	Dean and Lindley 2023
2021	11,232	0.87		9,785		
2022	16,383					
